# Direct conversion of methane to value-added hydrocarbons using alkali metal-promoted cobalt catalysts†

**DOI:** 10.1039/d5ra02408k

**Published:** 2025-07-07

**Authors:** Sarannuch Sringam, Punyanut Thansiriphat, Thongthai Witoon, Waleeporn Donphai, Metta Chareonpanich, Chularat Wattanakit, Hiesang Sohn, Nevzat Yigit, Günther Rupprechter, Anusorn Seubsai

**Affiliations:** a Department of Chemical Engineering, Faculty of Engineering, Kasetsart University Bangkok 10900 Thailand fengasn@ku.ac.th; b Center of Excellence on Petrochemical and Materials Technology, Kasetsart University Bangkok 10900 Thailand; c School of Energy Science and Engineering, Vidyasirimedhi Institute of Science and Technology Rayong 21210 Thailand; d Department of Chemical Engineering, Kwangwoon University Seoul 01897 South Korea; e Institute of Materials Chemistry, Technische Universität Wien Vienna 1060 Austria

## Abstract

The oxidative coupling of methane (OCM) is a promising pathway for directly converting methane into higher hydrocarbons (C_2+_). This research investigated the influence of alkali metal promoters (Li, Na, K, or Rb) on Co/Al_2_O_3_ catalysts prepared based on incipient wetness impregnation for the OCM reaction. The catalyst investigations demonstrated that the catalysts promoted with K and Rb had superior performance, with the 4.6K–Co/Al_2_O_3_ catalyst achieving a maximum C_2+_ yield of 8.1%, C_2+_ selectivity of 24.0%, and CH_4_ conversion of 32.1% at 640 °C. Catalyst characterization, based on XRD, HR-TEM, BET, XPS, CO_2_-TPD, and H_2_-TPR analyses, revealed the structural and physicochemical properties responsible for the enhanced catalytic activity. Specifically, K and Rb promoters increased surface basicity and enhanced the electron density of active sites, thereby promoting selective methane activation. *In-situ* DRIFTS and mechanistic studies highlighted the role of reactive oxygen species in promoting C_2+_ hydrocarbon formation. These results should position K–Co/Al_2_O_3_ as a promising catalyst for OCM and provide valuable guidance for designing more efficient catalytic systems for methane utilization.

## Abstract 2

This study explores alkali-promoted Co/Al_2_O_3_ catalysts for OCM. K- and Rb-promoted versions showed high C_2+_ yield and CH_4_ conversion at 490 °C. Enhanced basicity and active sites led to improved performance, aiding selective methane activation.

## Graphical abstract

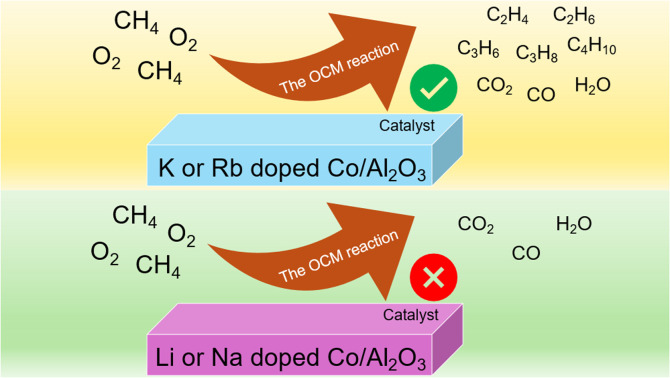

## 1. Introduction

Methane (CH_4_) and carbon dioxide (CO_2_) are the most potent greenhouse gases driving global warming and climate change.^1^ Methane, in particular, has a global warming potential approximately 25 times greater than CO_2_ over a 100 year period, making it a critical target for emission reduction and alternative utilization strategies.^2^ Despite its environmental impact, methane is also a valuable raw material for producing more complex and economically essential compounds. Efficient conversion of methane into higher-value chemicals can provide a dual benefit of mitigating climate impact and creating valuable products.^3^

Methane conversion can proceed through two primary pathways: indirect and direct.^4^ Indirect routes involve a two-step process, where methane is first reformed (*via* steam reforming, dry reforming, or partial oxidation) to produce syngas, a mixture of hydrogen (H_2_) and carbon monoxide (CO). Then, these syngas can be transformed into valuable chemicals, such as olefins and fuels, through processes such as Fischer–Tropsch synthesis. However, the indirect pathway is energy-intensive and requires multiple stages, driving interest toward more efficient direct conversion approaches.^5^

Direct methane conversion aims to simplify the process by producing valuable chemicals in a single step. Such methods include partial oxidation to formaldehyde and methanol or converting methane to higher hydrocarbons (C_2+_), such as ethylene (C_2_H_4_), ethane (C_2_H_6_), propylene (C_3_H_6_), propane (C_3_H_8_), and butanes (C_4_H_10_), *via* oxidative coupling of methane (OCM)^6^ or non-oxidative coupling of methane (NOCM). Although the NOCM process offers a promising route to convert methane without oxygen, it involves considerable thermodynamic challenges and requires high energy input, limiting its industrial viability.^7,8^ Consequently, OCM has attracted substantial attention as a feasible pathway for directly converting methane to C_2+_. In the OCM process, methane reacts with molecular oxygen at high temperatures (above 700 °C) to produce these valuable compounds and byproducts, including water, hydrogen, carbon monoxide, and carbon dioxide.^9^

Early research on OCM explored a range of catalysts, including pure oxides of rare earth, alkaline earth, and transition metals.^10^ However, the focus has shifted towards more sophisticated catalyst formulations to enhance methane conversion and selectivity toward higher hydrocarbons. Among the most extensively studied catalysts is Na_2_WO_4_–Mn/SiO_2_,^11,12^ which, despite its promising activity, has not been commercialized due to low C_2+_ yield and selectivity, coupled with issues of catalyst deactivation during prolonged operation.^13,14^ Challenges, such as sintering, phase changes, and coking, continue to limit the catalyst's industrial applicability, emphasizing the need for innovations that enhance performance and long-term stability.^6^

In 2023, we introduced a novel hybrid catalyst system for the direct conversion of CH_4_ to C_2+_, combining 15 wt% Ni supported on Al_2_O_3_ (15Ni/Al_2_O_3_) and 20 wt% Co supported on Al_2_O_3_ doped with 4.6 wt% K (4.6K–20Co/Al_2_O_3_). Operating at relatively low temperatures (490 °C), this catalyst demonstrated impressive results, achieving C_2+_ yields of 3.6–4.3%, with selectivity ranging from 7.9% to 15.8% and CH_4_ conversion rates between 27.2% and 46.3%.^15,16^ When compared to the individual catalysts under identical conditions, the hybrid catalyst outperformed both, showcasing the synergistic effect of combining Ni and K-promoted Co catalysts. Notably, the 4.6K–20Co/Al_2_O_3_ catalyst produced exceptionally high catalytic activity for methane conversion to C_2+_, whereas the unpromoted 20Co/Al_2_O_3_ catalyst was essentially inactive, yielding 0% C_2+_ products. This highlighted the crucial role of K as a promoter in facilitating the direct activation of CH_4_—a finding that warranted further investigation.

The selection of appropriate promoters is crucial for addressing the limitations of traditional OCM catalysts. When integrated into the catalyst, alkali metals function as modifiers that enhance the surface basicity.^17^ This modulation of catalytic properties can redirect reaction pathways, promoting the formation of higher hydrocarbons (C_2+_).^18^ However, comprehensive studies that have systematically compared the effects of various alkali metals on cobalt-based catalysts have not been explored. In refining catalyst designs, it is crucial to understand how different alkali metal promoters influence the catalyst structure, activity, and selectivity.

Given that K belongs to the alkali metals group, which also includes lithium (Li), sodium (Na), rubidium (Rb), and cesium (Cs), it raises the intriguing possibility that other alkali metals may produce similar effects when used as promoters. Therefore, in this study, we explored the influence of various viable alkali metals (Li, Na, K, and Rb) on the performance of 20Co/Al_2_O_3_ catalysts in the OCM reaction. We systematically investigated how these promoters impacted catalytic activity, product selectivity, and CH_4_ conversion. In addition, we examined the effect of metal loading on optimizing the OCM process. Various advanced characterization techniques were applied to understand the relationships between the physical and chemical properties of the catalysts and their performance, offering insights into the design of more efficient and stable catalysts for direct methane conversion.

## 2. Experimental

### 2.1 Catalyst preparation

All the catalysts were prepared using the incipient wetness impregnation method. The Co/Al_2_O_3_ catalyst was promoted with different weights of four alkali metals: Li, Na, K, and Rb. Several metal nitrates were used as precursors, consisting of LiNO_3_ (99.99%, Sigma-Aldrich), NaNO_3_ (99.5%, Alfa Aesar), RbNO_3_ (99%, Alfa Aesar), KNO_3_ (99%, Alfa Aesar), and Co (NO_3_)_2_·6H_2_O (99%, Alfa Aesar). The support used for all catalysts was γ-Al_2_O_3_ (denoted as Al_2_O_3_, with a surface area of 75.32 m^2^ g^−1^, 99.97%, Alfa Aesar). Each metal precursor was dissolved in deionized water as a stock solution in the first step. Then, each solution was dropped onto Al_2_O_3_. Each mixture was stirred for 1 hour at room temperature before heating and continuously stirring at 90 °C until dry. Each dried sample was ground and calcined at 400 °C for 1 hour at a heating rate of 10 °C min^−1^. The weight percentage of Co in all catalysts was fixed at 20, while the weight percentage of each promoter was in the range 0.1–10.0, with the balance comprising the weight percentage of the Al_2_O_3_ support. For example, one catalyst was denoted as 4.6K–Co/Al_2_O_3_, representing 4.6wt% K, 20wt% Co, and 75.4wt% Al_2_O_3_. Thus, there were five catalyst groups studied: Li–Co/Al_2_O_3_, Na–Co/Al_2_O_3_, K–Co/Al_2_O_3_, Rb–Co/Al_2_O_3_, and Co/Al_2_O_3_.

### 2.2 Catalyst characterization

The crystalline structure of each sample was identified using X-ray diffractometry (XRD; Rigaku Smart Lab XE, 9 kW), using Cu-Kα radiation at 40 kV and 100 mA, a step size of 0.01°, a scan speed of 3° min^−1^, and a 2*θ* range of 10–80°.

The morphology of the samples was observed using high-resolution transmission electron microscopy (HR-TEM; JEM-ARM200F) and a high-angle annular dark-field (HAADF) scanning transmission electron microscopy (TEM) and energy dispersive X-ray spectrometry (EDS) (JEM-ARM200F). The operating voltage for the TEM was 200 kV. Before analysis, each sample was prepared by dispersing it in an ethanol solution for 30 min and dropping it onto a copper TEM grid. Then, it was dried in a chamber filled with nitrogen at room temperature.

The surface area, pore volume, and pore size of each catalyst was determined using a nitrogen-physisorption analyzer (3Flex Physisorption Micrometrics). Before measurement, each sample was degassed overnight at 200 °C to remove moisture and other adsorbed molecules. The Brunauer–Emmett–Teller model was used to calculate the surface area, while the Barrett–Joyner–Halenda model was used to calculate the pore size distribution.

The binding energy of Co in each catalyst was analyzed using X-ray photoelectron spectroscopy (XPS; Kratos Model Axis ultra DLD), with a monochromator (Al Kα) as the X-ray source and beam current of 10 mA, with a voltage of 15 kV. The spectra of Co 2p were collected at a pass energy of 40 eV in steps of 0.1 eV. All spectra were calibrated using the C1s signal of the carbon support material at 284.6 eV.

The surface basicity of the catalyst was analyzed using temperature-programmed desorption of carbon dioxide (CO_2_-TPD) using an AutoChem II 2920 (Micromeritics). Each sample (200 mg) was contained in a quartz U-tube and heated to 400 °C under a flow of helium (He) gas for 30 min and cooled to 200 °C. Subsequently, a flow of 10% CO_2_ in He gas was applied for 60 min and then purged with He gas until the baseline was stable. Next, it was heated again from 200 to 800 °C (heating rate of 10 °C min^−1^), and the CO_2_ desorbed was detected using a thermal conductivity detector (TCD).

The reducibility of the catalysts was analyzed using temperature-programmed reduction of hydrogen (H_2_-TPR) using an AutoChem II 2920 (Micromeritics). Each sample (200 mg) was contained in a quartz U-tube and heated to 150 °C under a flow of argon (Ar) gas for 30 min and cooled to 50 °C. When the baseline was stable, a flow of 10% H_2_ in Ar gas was applied, and the temperature was increased to 1000 °C (heating rate of 5 °C min^−1^). The quantity of H_2_ consumption was detected using a TCD.

Thermogravimetry/differential thermal analysis (TG/DTA, PerkinElmer TGA 8000) was performed under atmospheric pressure. Prior to analysis, the samples were dried at 80 °C overnight to eliminate residual moisture. TG/DTA measurements were conducted over a temperature range of 30–800 °C, using a heating rate of 5 °C min^−1^ and N_2_ flow rate of 50 mL min^−1^.

A single-beam infrared spectrometer (Bruker VERTEX 70v FT-IR) coupled with a wide-band mercury-cadmium-telluride detector and a liquid-nitrogen-cooled system was used to perform *in situ* diffuse reflectance infrared Fourier transform spectroscopy (DRIFTS). The catalyst (20 mg) was placed inside a stainless-steel flow cell oven with a CaF_2_ window. The catalyst was pretreated at 400 °C under Ar gas with a flow rate of 40 mL min^−1^ for 1 hour, followed by cooling to room temperature. Subsequently, the gas was converted to a CH_4_ : O_2_ : N_2_ mixture gas with a 2 : 1 : 4 ratio and a total flow rate of 50 mL min^−1^, and the temperature was increased to 490 °C at a heating rate of 10 °C min^−1^, with the spectrum being recorded at 1 min intervals for 30 min. Each spectrum was collected based on 128 scans at a resolution of 4 cm^−1^ over a spectral range of 900–4000 cm^−1^.

### 2.3 Catalyst activity testing

Each catalyst (40 mg) was packed between quartz wool in a quartz tube with a diameter of 0.5 cm in a plug flow reactor. The reactant gases, consisting of CH_4_ (99.999%, Labgaz) and O_2_ (99.999%, Linde), with a CH_4_ : O_2_ ratio of 2 and a total flow rate of 40 mL min^−1^, were fed to the quartz tube at atmospheric pressure and a reaction temperature of 440−740 °C. The feed gases were controlled using mass flow controllers (Aalborg GFC17). The effluent gases were analyzed using an online gas chromatograph (GC-14A; Shimadzu) equipped with a flame ionization detector to evaluate C_2_H_4_, C_2_H_6_, C_3_H_6_, C_3_H_8_, C_4_H_8_, and C_4_H_10_ and a TCD was used to assess CO, CO_2_, and CH_4_. A standard calibration curve of five calibration points was established for each gas, with an *R*-squared value exceeding 0.995. This enabled accurate quantification of the mole of effluent gas. The activity of each catalyst was expressed as %CH_4_ conversion, %C_2+_ selectivity, and %C_2+_ yield, as shown in eqn (1)–(3), respectively.1
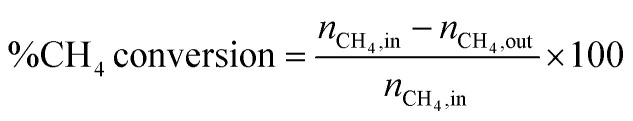
2
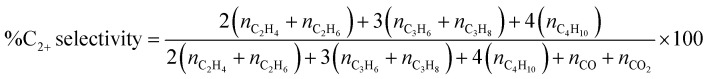
3
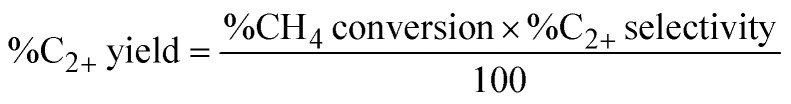
where *n* is the number of moles.

Each catalyst testing study was conducted at least three times, and the results were repeatable within 10%. The data were presented as average values except for the catalyst stability test. Furthermore, the catalytic performance data contained less than 5% carbon balance errors.

## 3. Results and discussion

### 3.1 Performance of catalysts

**Fig. 1:**
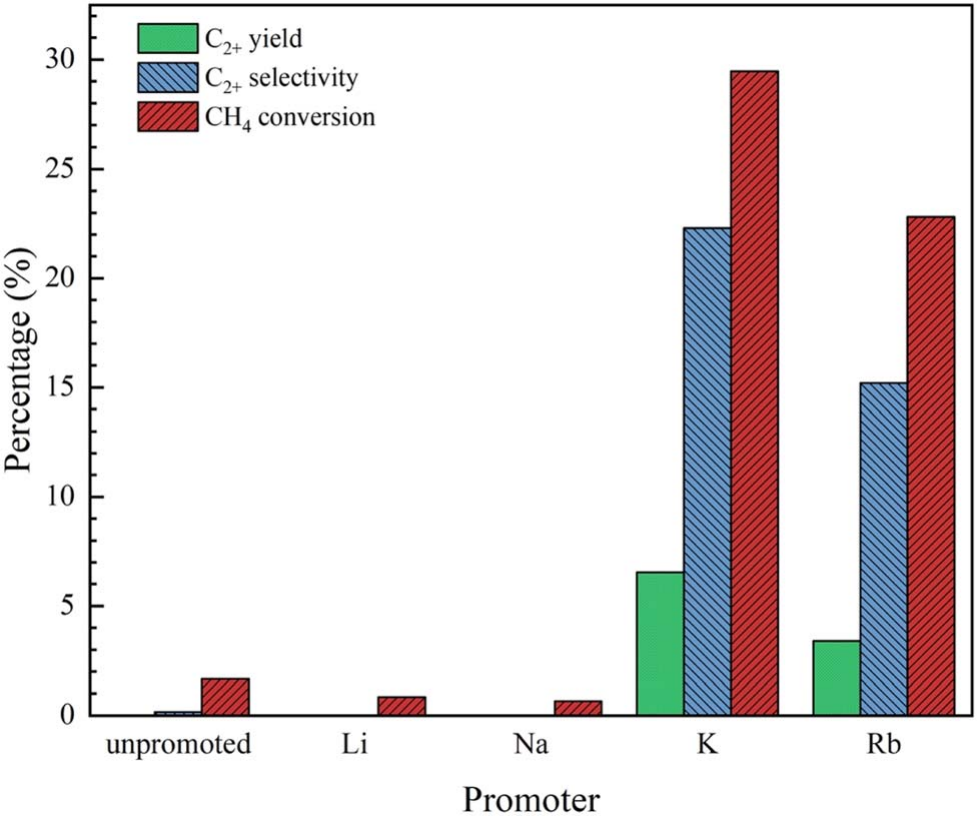
Catalytic performance of Co/Al_2_O_3_ catalysts with different promoters for OCM reaction. Reaction conditions: CH_4_ : O_2_ ratio = 2 : 1, catalyst weight = 40 mg, total feed flow rate = 40 mL min^−1^, reactor temperature = 490 °C.

The catalytic performance levels of the 20 wt% Co/Al_2_O_3_ catalysts, both unpromoted and promoted with 4.6 wt% of alkali metals (Li, Na, K, and Rb), were evaluated in a plug flow reactor under reaction conditions of 490 °C and atmospheric pressure. As depicted in Fig. 1, incorporating the different alkali promoters led to considerable variations in catalytic activity, allowing the catalysts to be categorized into two distinct groups based on their performance. Group I comprised 4.6K–Co/Al_2_O_3_ and 4.6Rb–Co/Al_2_O_3_, which had superior catalytic activity, while Group II included 4.6Li–Co/Al_2_O_3_, 4.6Na–Co/Al_2_O_3_, and the unpromoted Co/Al_2_O_3_, all of which had comparatively lower performance.

The catalysts in Group I achieved C_2+_ hydrocarbon yields in the range 3.4–6.5%, with C_2+_ selectivity in the range 15.2–22.3% and CH_4_ conversion rates in the range 22.8–29.5%. These results highlighted the enhanced catalytic behavior when K or Rb was used as a promoter. In contrast, the Group II catalysts produced negligible C_2+_ yields (0%) and minimal C_2+_ selectivity (0–0.1%), with CH_4_ conversion rates limited to 0.7–1.7%. This stark difference from Group 1 underscored the effectiveness of the K and Rb promoters in enhancing the activity of Co/Al_2_O_3_ catalysts compared to Li, Na, or no promoter at all.

Based on the results, it was clear that the 4.6K–Co/Al_2_O_3_ and 4.6Rb–Co/Al_2_O_3_ catalysts outperformed their counterparts, making them the most promising candidates for further investigation. Consequently, these two catalysts were selected for detailed characterization, as described in Section 3.2, to elucidate the reasons behind their superior catalytic behavior. For comparative purposes, the unpromoted Co/Al_2_O_3_ catalyst was also characterized to provide a comprehensive analysis of the effects of alkali promotion.

### 3.2 Catalyst characteristics

#### 3.2.1 Crystal structures of catalyst

**Fig. 2:**
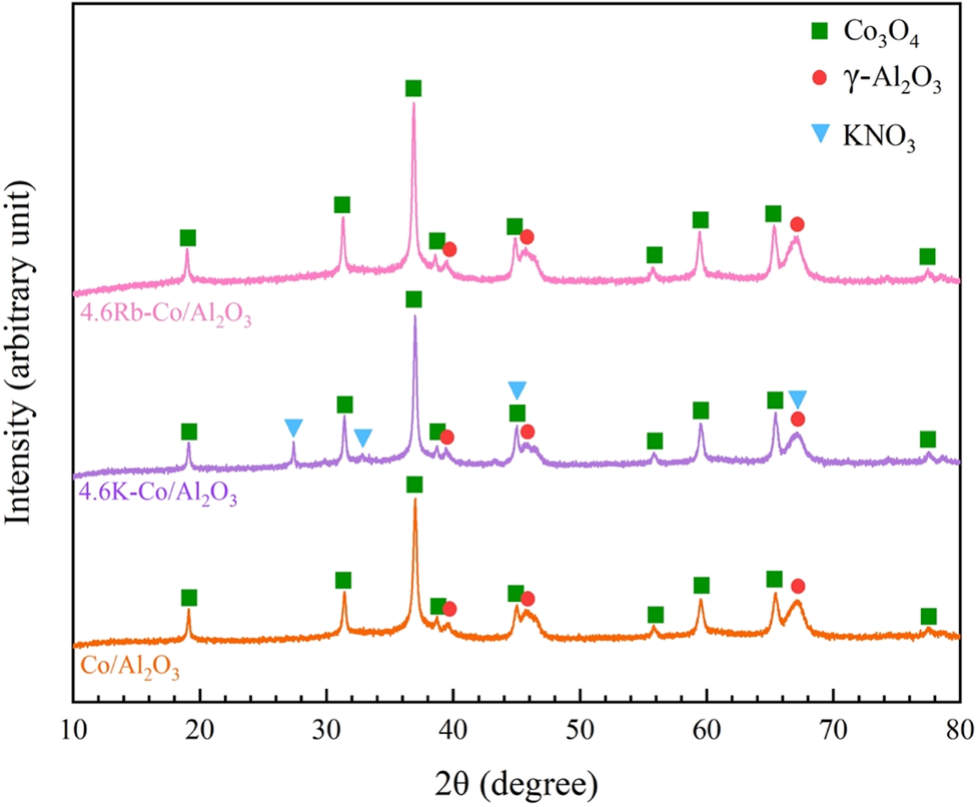
XRD patterns of Co/Al_2_O_3_, 4.6K–Co/Al_2_O_3_, and 4.6Rb–Co/Al_2_O_3_ catalysts.

The catalysts—Co/Al_2_O_3_, 4.6K–Co/Al_2_O_3_, and 4.6Rb–Co/Al_2_O_3_—were rigorously analyzed using XRD to identify their distinct crystalline phases. Fig. 2 presents the XRD patterns of each catalyst, with the detailed phase information tabulated in Table S1.† Notably, the γ-Al_2_O_3_ phase appeared consistently across all catalysts, attributed to the identical preparation conditions. Furthermore, for all samples calcined above 250 °C, Co_3_O_4_ crystallinity was evident, aligning with the known decomposition temperature of Co(NO_3_)_2_ to Co_3_O_4_.^19^ Intriguingly, XRD patterns of the 4.6K–Co/Al_2_O_3_ catalyst revealed a crystalline KNO_3_ phase rather than K_2_O, since the transformation to K_2_O occurs only beyond 650 °C.^20^ In contrast, the 4.6Rb–Co/Al_2_O_3_ catalyst lacked a discernible crystalline of Rb species, likely due to its amorphous nature or the undetectable crystal size within the XRD sensitivity range.

#### 3.2.2 Catalyst morphology

**Fig. 3:**
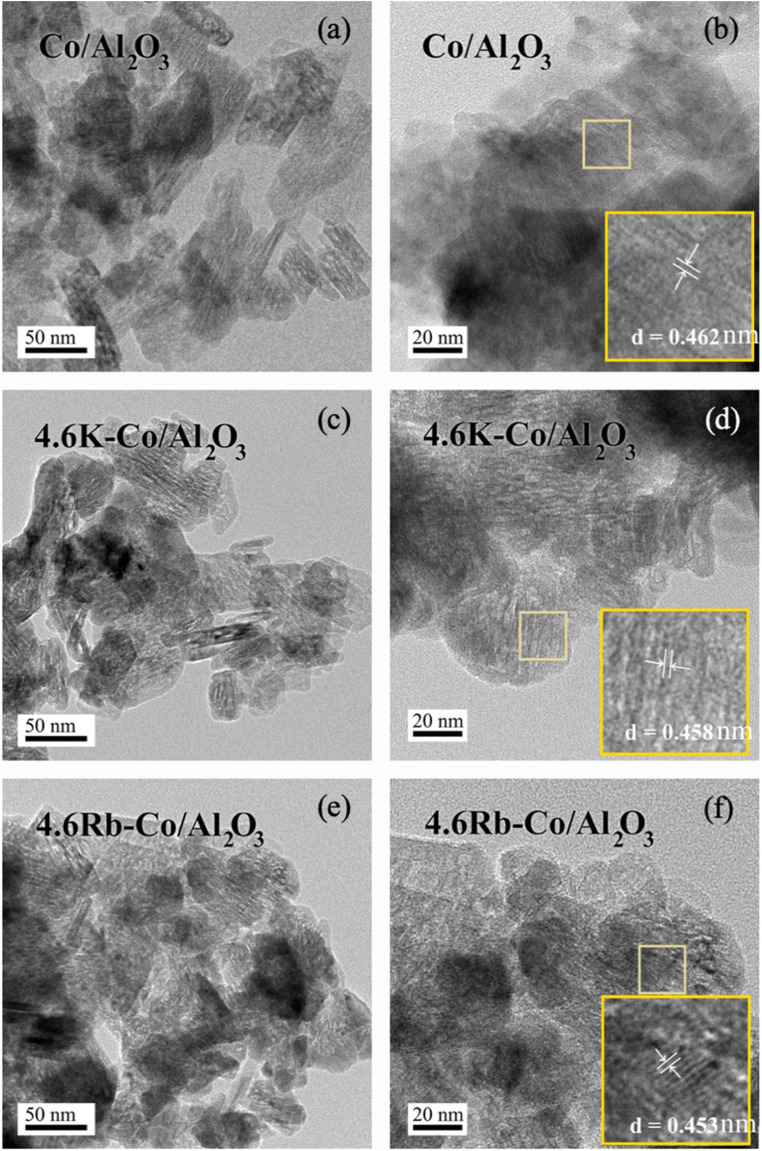
HR–TEM images of (a and b) Co/Al_2_O_3_, (c and d) 4.6K–Co/Al_2_O_3_, and (e and f) 4.6Rb–Co/Al_2_O_3_.

As illustrated in Fig. 3, the HR-TEM results provided an in-depth look at the morphology of the Co/Al_2_O_3_, 4.6K–Co/Al_2_O_3_, and 4.6Rb–Co/Al_2_O_3_ catalysts. The catalyst particles displayed a variety of irregular shapes and sizes, with dimensions consistently in the range 38–41 nm (see particle size distribution in Fig. S1†). Across all catalysts, the Co_3_O_4_ particles were dispersed uniformly on the Al_2_O_3_ support, with the Co_3_O_4_ (111) crystalline phase having an average *d*-spacing of 0.453–0.462 nm.^21^

**Fig. 4:**
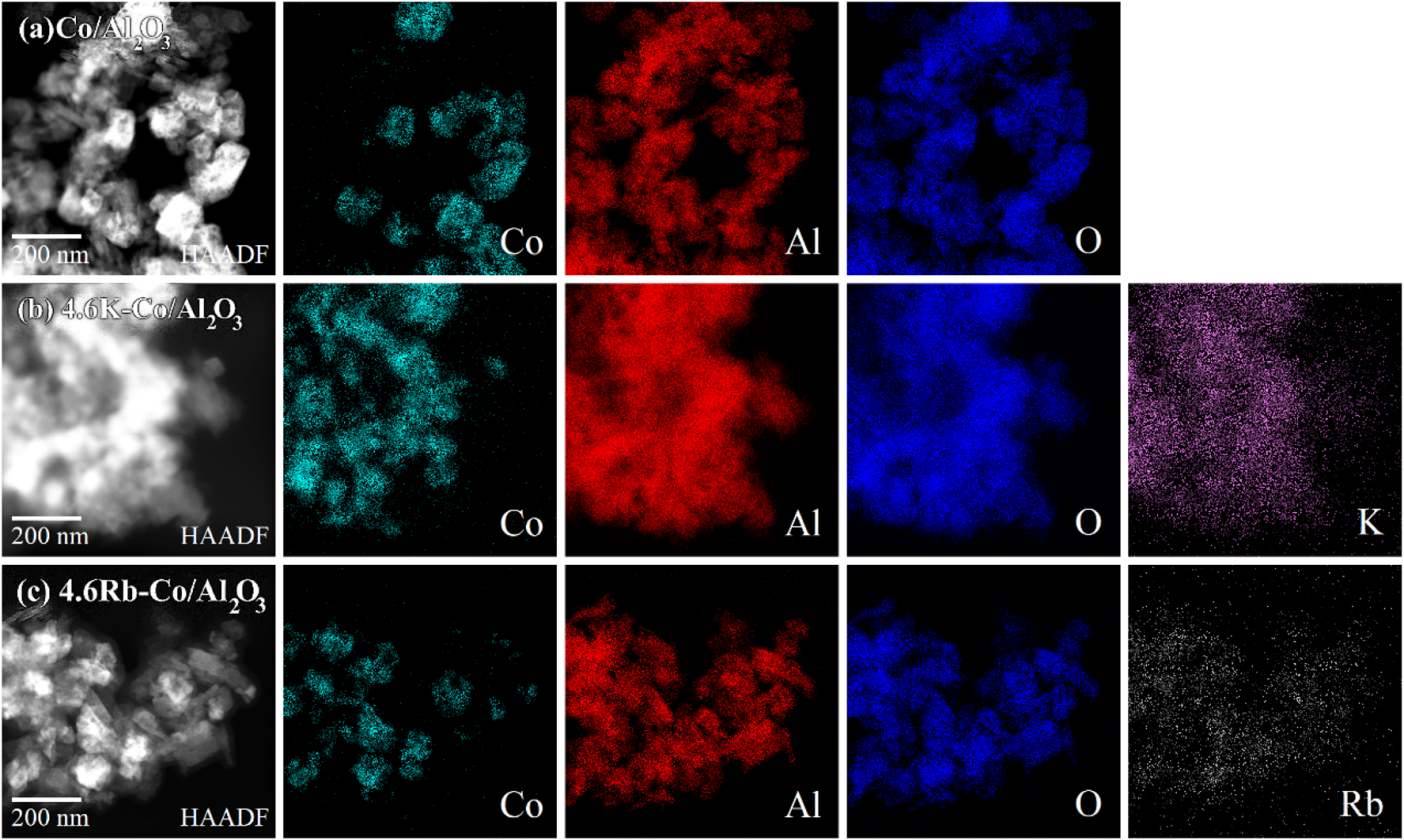
Images of HAADF with EDS elemental mapping of (a) Co/Al_2_O_3_, (b) 4.6K–Co/Al_2_O_3_, and (c) 4.6Rb–Co/Al_2_O_3_.

In Fig. 4, the HAADF images with EDS elemental mapping reveal a robust elemental distribution of Co, Al, O, K, and Rb. These elements were distributed across all catalysts. Notably, while the crystalline phase of Rb species was undetected in XRD, the HAADF-EDS images distinctly indicate the dispersion of Rb. The even elemental distribution—particularly of the oxygen species—is advantageous for catalytic efficiency in OCM processes.^22^

#### 3.2.3 Physical properties of catalyst

**Fig. 5:**
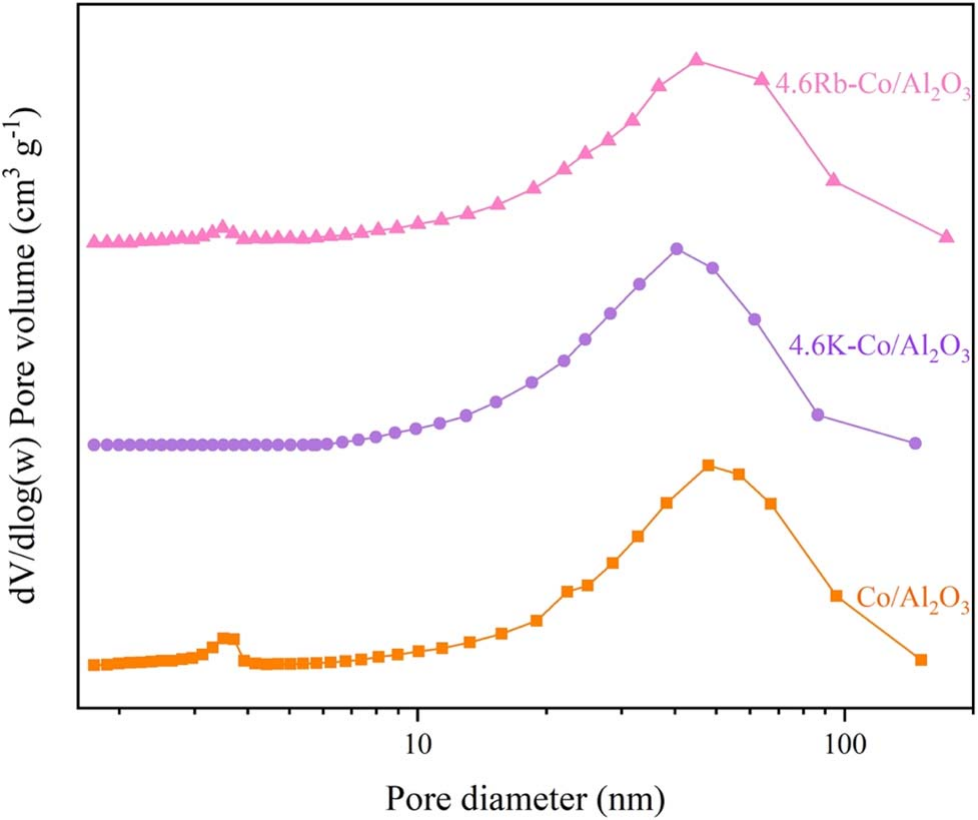
Pore size distribution of Co/Al_2_O_3_, 4.6K–Co/Al_2_O_3_, and 4.6Rb–Co/Al_2_O_3_ catalysts.

**Fig. 6:**
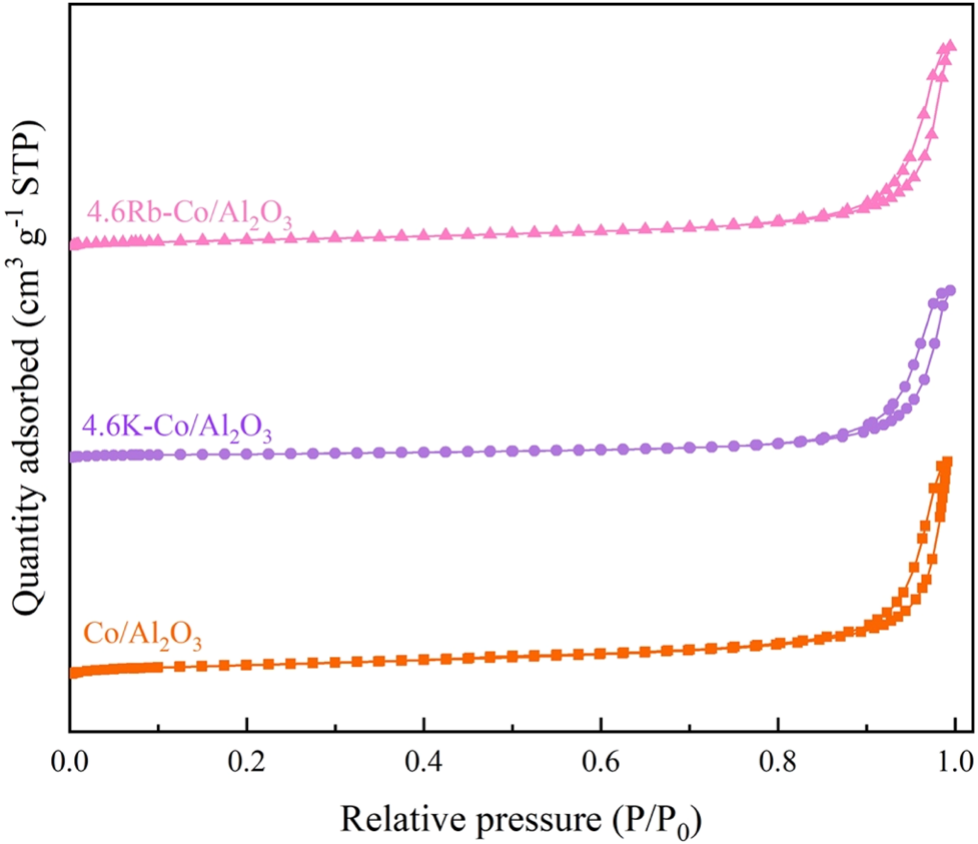
N_2_ adsorption–desorption isotherms of Co/Al_2_O_3_, 4.6K–Co/Al_2_O_3_, and 4.6Rb–Co/Al_2_O_3_ catalysts.

Table 1 summarizes the physical parameters of the catalyst surfaces determined using the N_2_-sorption technique. The Co/Al_2_O_3_ catalyst had a surface area of 60.39 m^2^ g^−1^ and a pore volume of 0.34 cm^3^ g^−1^. After impregnation with K and Rb, these surface properties were reduced due to the deposition of K and Rb within the Al_2_O_3_ pores. Specifically, the surface area and pore volume of the 4.6K–Co/Al_2_O_3_ catalyst decreased to 27.80 m^2^ g^−1^ and 0.27 cm^3^ g^−1^, respectively, and for the 4.6Rb–Co/Al_2_O_3_ catalyst to 46.40 m^2^ g^−1^ and 0.32 cm^3^ g^−1^, respectively. Additionally, Fig. 5 illustrates the variation in pore size. The Co/Al_2_O_3_ and 4.6Rb–Co/Al_2_O_3_ catalysts were a bimodal porous material, but the 4.6K–Co/Al_2_O_3_ was a monomodal porous material, which may occur as a deposit of metal oxides inside the small pores, leading to the loss of one of the pore size regimes. The mean pore diameters of the 4.6K–Co/Al_2_O_3_ (40.46 nm) and 4.6Rb–Co/Al_2_O_3_ catalysts (3.49 nm and 44.89 nm) were smaller than that of the Co/Al_2_O_3_ catalyst (3.71 nm and 47.90 nm), which could be attributed to the presence of K and Rb within the catalyst pores. Furthermore, the N_2_ adsorption–desorption isotherm of the catalysts is shown in Fig. 6. According to the International Union of Pure and Applied Chemistry classification, the catalysts exhibited Type IV adsorption isotherms with an H3 hysteresis loop, suggesting that all catalysts were mesoporous materials.^23^

**Table 1:** Surface area, pore volume, and pore diameter of each catalyst

Catalyst	Surface area (m^2^ g^−1^)	Pore volume (cm^3^ g^−1^)	Pore diameter (nm)
Co/Al_2_O_3_	1.21, 59.18	0.0001, 0.34	3.71, 47.90
4.6K–Co/Al_2_O_3_	27.80	0.27	40.46
4.6Rb–Co/Al_2_O_3_	4.90, 41.50	0.002,0.32	3.49, 44.89

#### 3.2.4. Chemical state of catalysts

**Fig. 7:**
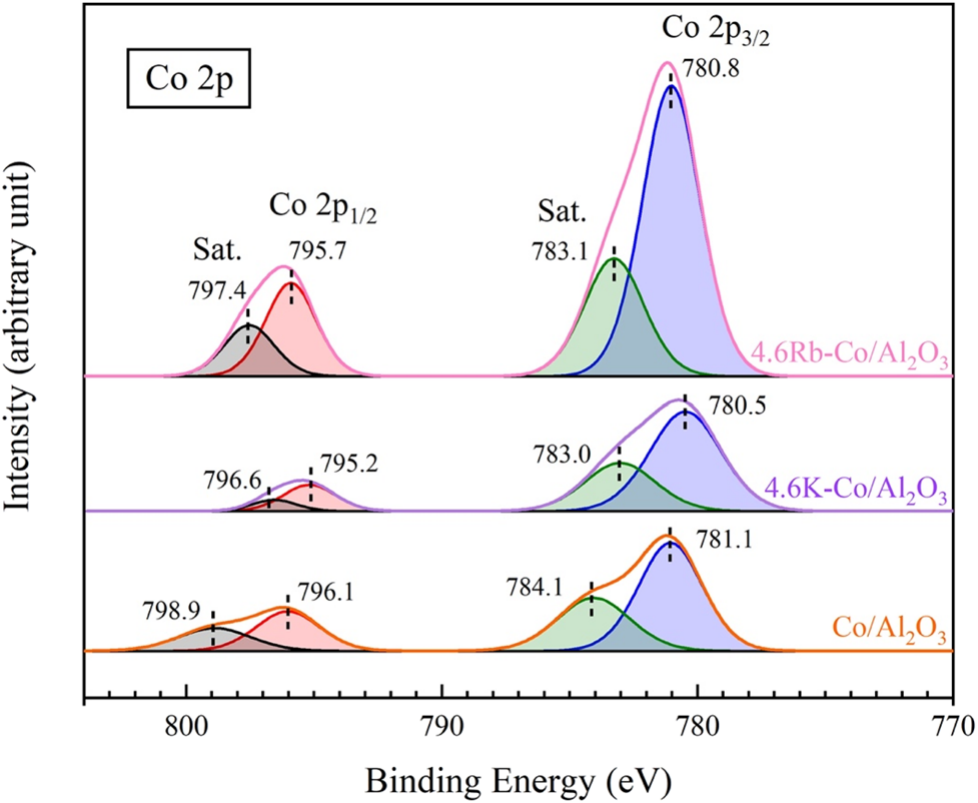
XPS spectra of Co/Al_2_O_3_, 4.6K–Co/Al_2_O_3_, and 4.6Rb–Co/Al_2_O_3_ catalysts.

Fig. 7 presents the XPS spectra in the Co 2p regions for the catalysts. The Co 2p spectra for all catalysts displayed two distinct regions: at Co 2p_3/2_ at lower binding energies (775–790 eV) and Co 2p_1/2_ at higher binding energies (790–803 eV), which are characteristic of the Co_3_O_4_ phase^24^ and aligned with the XRD results, confirming the presence of Co_3_O_4_ in the composites. For the Co/Al_2_O_3_ catalyst, two peaks were observed at approximately 781.1 and 796.1 eV, corresponding to Co 2p_3/2_ and Co 2p_1/2_, respectively, while the two peaks at approximately 784.1 and 798.9 eV were their satellite peaks.^25^ With the impregnation of Co/Al_2_O_3_ with K and Rb, the binding energies of both Co 2p_3/2_ and Co 2p_1/2_ peaks shifted to lower values, reflecting alterations in the catalyst's electronic environment and surface chemistry. This decrease in binding energy likely resulted from the promoters donating electron density to active metal sites or modifying the surface structure, thereby influencing the electron distribution around the atoms.^26^

For the 4.6K–Co/Al_2_O_3_ and 4.6Rb–Co/Al_2_O_3_ catalysts, there were shifts to lower binding energy values for both Co 2p_3/2_ and Co 2p_1/2_ compared to the catalyst without the dopant. The binding energy of each catalyst is summarized in Table S2.† These shifts to lower binding energies in the XPS spectra suggested an increase in electron density around the active sites, which promoted selective methane activation while reducing the likelihood of complete oxidation to CO and CO_2_.^27^ Consequently, the 4.6K–Co/Al_2_O_3_ catalyst showed better selectivity for C_2_ hydrocarbons than the 4.6Rb–Co/Al_2_O_3_ catalyst.

#### 3.2.5. Surface basicity of catalyst

**Fig. 8:**
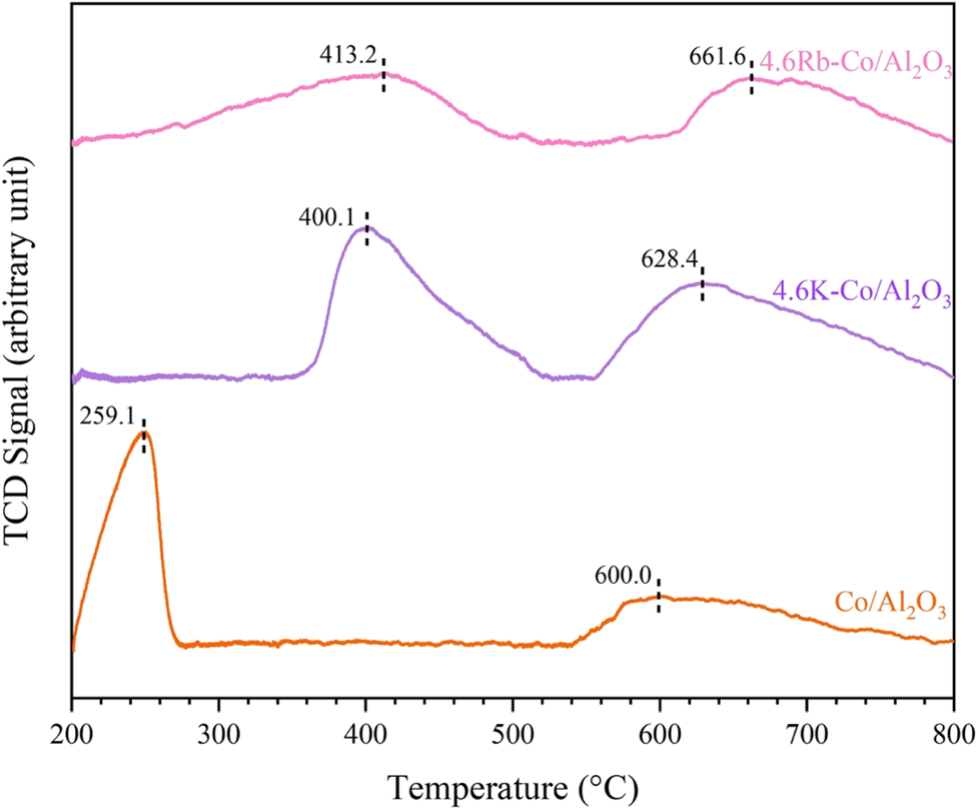
CO_2_-TPD profiles of Co/Al_2_O_3_, 4.6K–Co/Al_2_O_3_, and 4.6Rb–Co/Al_2_O_3_ catalysts.

The basicity of the catalysts was evaluated using CO_2_-TPD desorption profiles over a temperature range of 200–800 °C, as shown in Fig. 8. The CO_2_-TPD profiles for each catalyst could be divided into two categories: moderate basic sites (200–540 °C) and strong basic sites (560–800 °C). The desorption peaks for the Co/Al_2_O_3_, 4.6K–Co/Al_2_O_3_, and 4.6Rb–Co/Al_2_O_3_ catalysts were observed at 256.2, 400.1, and 413.2 °C, respectively, in the moderate temperature range, and at 600.0, 628.4, and 661.6 °C, respectively, in the strong temperature range. Surface basic sites, especially moderate basic sites, facilitate the adsorption and activation of CH_4_ molecules, resulting in the formation of methyl radicals essential for C_2_ hydrocarbon production.^5^ Therefore, C_2_ selectivity in the OCM reaction correlates with the estimated quantity of moderate basic sites, reflected by the area under the CO_2_-TPD curve,^28,29^ as summarized in Table 2. Compared to the Co/Al_2_O_3_ catalyst, the 4.6K–Co/Al_2_O_3_, and 4.6Rb–Co/Al_2_O_3_ catalysts had notable increases in the concentration of their moderate basic sites, attributed to the enhanced electron density on the surface introduced by these promoters, which effectively increased the overall basicity. The Co/Al_2_O_3_ catalyst had the lowest concentration of moderate basic sites, whereas the 4.6K–Co/Al_2_O_3_ catalyst had the highest concentration. This distribution was consistent with the catalytic performance results presented in Fig. 1, suggesting that the addition of K to the Co/Al_2_O_3_ catalyst enhanced the concentration of moderate basic sites, thereby facilitating the abstraction of H from CH_4_ to form CH_3_—an essential intermediate in the catalytic OCM reaction.^30^

**Table 2:** Distribution of the strength of basicity of catalysts

Catalyst	Basicity amount (μmol g^−1^)	
	Moderate basic sites (200–540 °C)	Strong basic sites (540–800 °C)
Co/Al_2_O_3_	0.83	0.63
4.6K–Co/Al_2_O_3_	1.37	1.22
4.6Rb–Co/Al_2_O_3_	0.98	0.72

#### 3.2.6. Reduction properties of catalysts

**Fig. 9:**
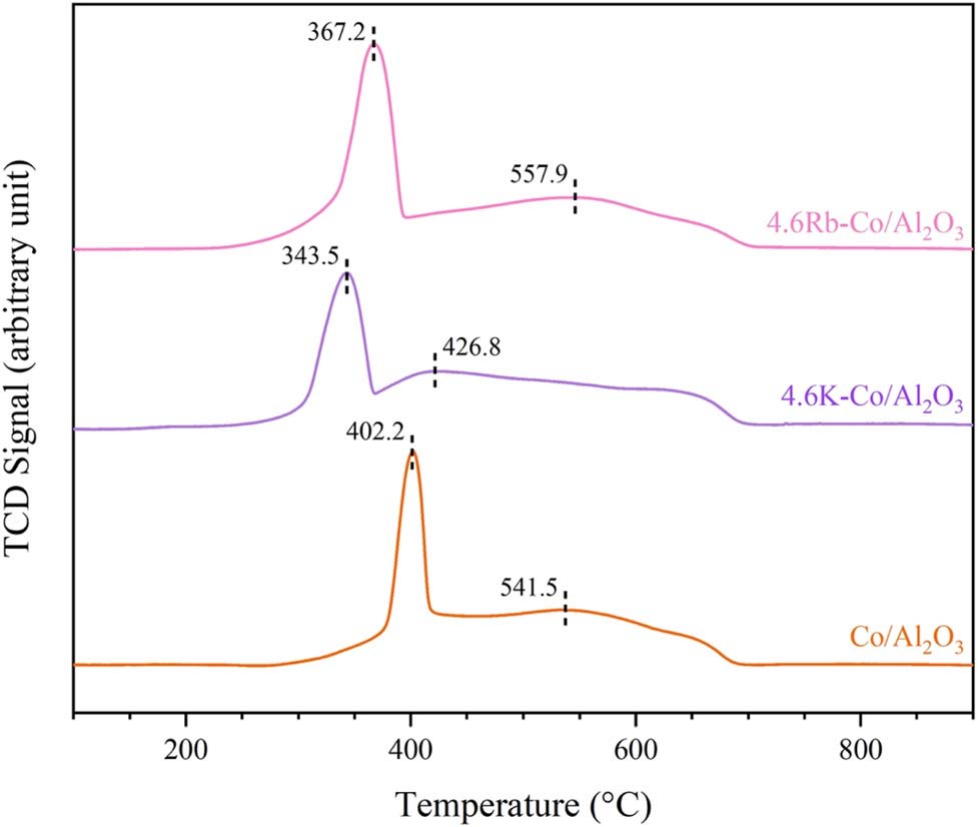
H_2_-TPR profiles of Co/Al_2_O_3_, 4.6K–Co/Al_2_O_3_, and 4.6Rb–Co/Al_2_O_3_ catalysts.

The reducibility of the catalysts was assessed using H_2_-TPR analysis, as shown in Fig. 9. Generally, the reduction of Co_3_O_4_ to metallic Co proceeds through two steps (Co_3_O_4_ → CoO → Co^o^), where the reduction of Co_3_O_4_ generally takes place between 250 and 400 °C, followed by the reduction of CoO in the range of 400–600 °C.^31^ In the current study, the H_2_-TPR profiles for all catalysts displayed two reduction peaks: the first, corresponding to the reduction of Co_3_O_4_ to CoO, occurred at 402.2 °C, 343.5 °C, and 367.3 °C for the Co/Al_2_O_3_, 4.6K–Co/Al_2_O_3_, and 4.6Rb–Co/Al_2_O_3_ catalysts, respectively. The second peak, associated with reducing CoO to Co, appeared at 541.5 °C, 426.8 °C, and 557.9 °C for these catalysts, respectively. These results demonstrated that adding the promoters to the Co/Al_2_O_3_ catalyst impacted their reduction temperatures. The 4.6K–Co/Al_2_O_3_ catalyst had the lowest reduction temperatures, indicating the presence of highly reducible species and suggesting that the oxygen species could be replenished rapidly on the catalyst surface, ensuring a steady supply of reactive oxygen species (ROS) essential for methane activation.^32^ Such a characteristic is crucial for sustaining catalytic activity and enhancing methane conversion efficiency. These findings aligned with the catalytic performance results, as the 4.6K–Co/Al_2_O_3_ catalyst had the highest activity among all the tested catalysts.

### 3.3 Optimal promoter weight percentage

**Fig. 10:**
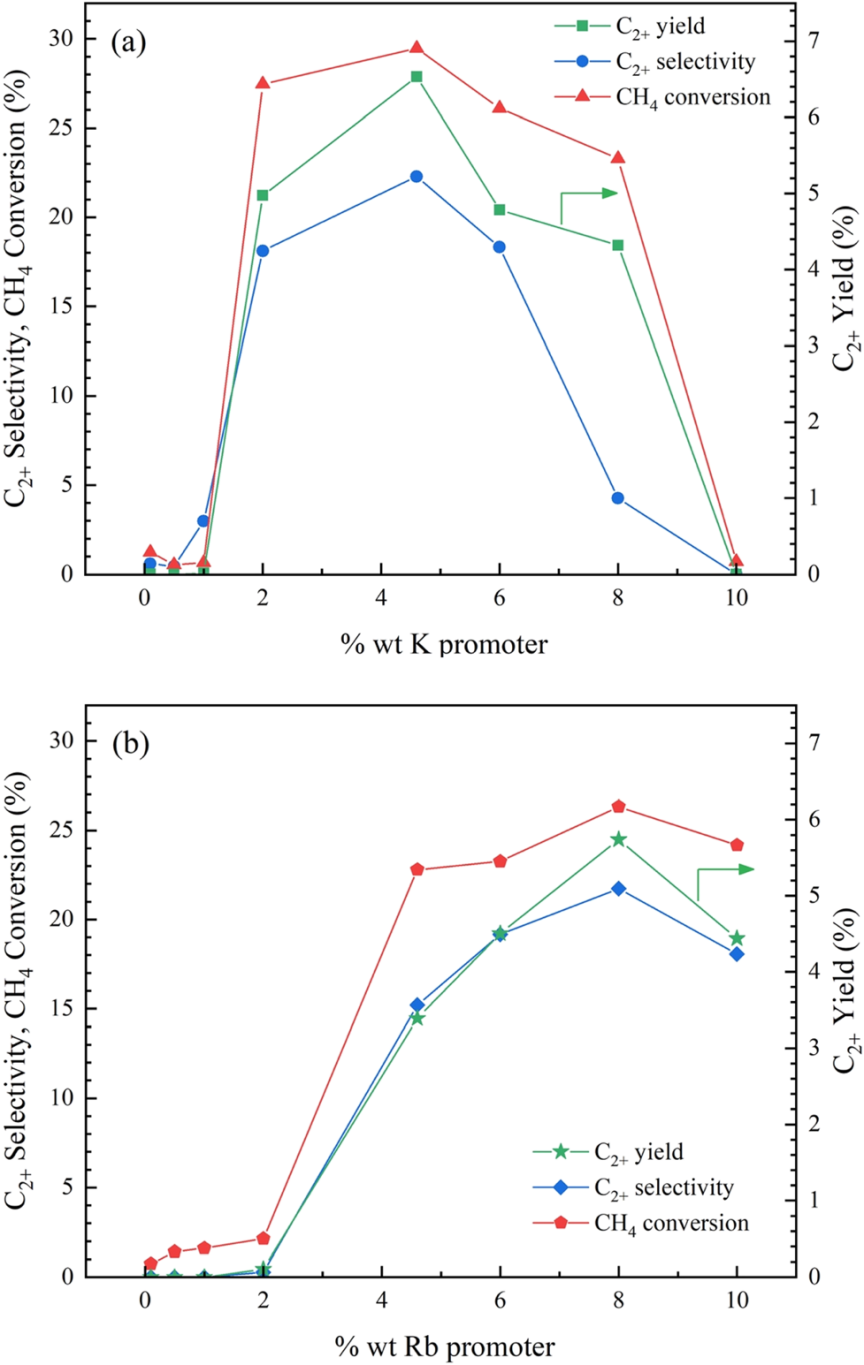
Catalytic performance of (a) K–Co/Al_2_O_3_ catalyst and (b) Rb–Co/Al_2_O_3_ with varying weight percentages of promoter for OCM reaction. Reaction conditions: CH_4_ : O_2_ ratio = 2 : 1, catalyst weight = 40 mg, total feed flow rate = 40 mL min^−1^, reactor temperature = 490 °C.

According to Section 3.1, the Co/Al_2_O_3_ catalyst doped with K and Rb had high activity for the OCM reaction. This section describes the testing of different loadings (0.1, 0.5, 1.0, 2.0, 4.6, 6.0, 8.0, and 10.0 wt%) of these two promoters on the catalyst for the OCM reaction at atmospheric pressure and the reaction temperature of 490 °C. The activity results are shown in Fig. 10. The C_2+_ formation could be seen for the catalysts doped with K (0–0.5wt%) and Rb (0–2.0wt%). Then, the levels of catalytic performance (C_2+_ yield, C_2+_ selectivity, and CH_4_ conversion) increased with an increasing percentage of promoters because the promoters formed active sites essential for methane activation and the ensuing coupling processes.^33^ The highest performance percentages were 6.5% C_2+_ yields, 22.3% C_2+_ selectivity, and 29.5% CH_4_ conversion for the 4.6K–Co/Al_2_O_3_ catalyst and 5.7% C_2+_ yield, 21.7% C_2+_ selectivity, and 26.3% CH_4_ conversion for the 8Rb–Co/Al_2_O_3_ catalyst. However, excessive promoter loading resulted in aggregation or inadequate dispersion of active sites, reducing the effective surface area available for the reaction and potentially decreasing catalytic activity.^34^ These testing results indicated that the most effective catalyst was the 4.6K–Co/Al_2_O_3_ catalyst.

### 3.4 Optimal reaction temperature

**Fig. 11:**
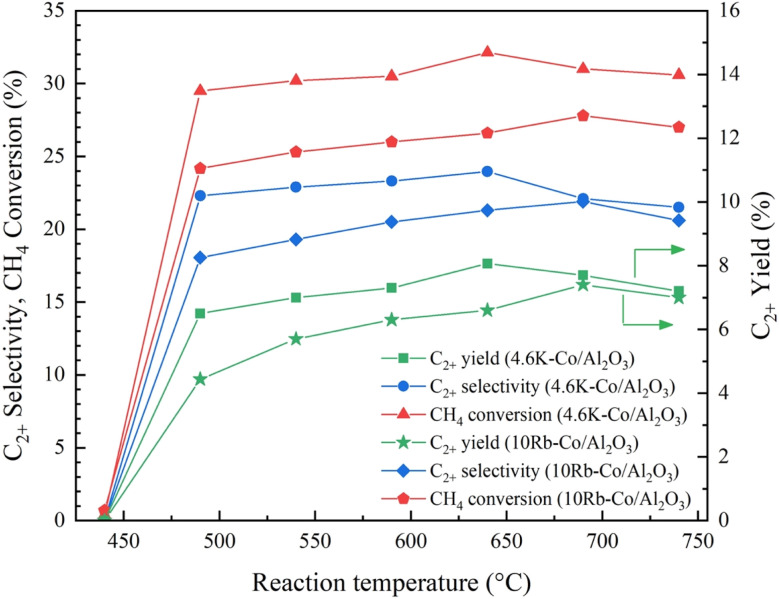
Catalytic performance of the 4.6K–Co/Al_2_O_3_ and 10Rb–Co/Al_2_O_3_ catalyst with varying reaction temperatures for OCM reaction. Reaction conditions: CH_4_ : O_2_ ratio = 2 : 1, catalyst weight = 40 mg, total feed flow rate = 40 mL min^−1^, reactor temperature = 440–740 °C.

To ensure a fair comparison based on the alkali metal content, the two catalysts were reformulated to possess equivalent molar amounts of potassium and rubidium. This adjustment resulted in catalysts with revised weight loadings: 4.6K–Co/Al_2_O_3_ (*i.e.*, 2.05 molar of K on Co/Al_2_O_3_) and 10Rb–Co/Al_2_O_3_ (*i.e.*, 2.05 molar of Rb on Co/Al_2_O_3_). The impact of reaction temperature on catalytic performance was examined for both 4.6K–Co/Al_2_O_3_ and 10Rb–Co/Al_2_O_3_ catalysts over a temperature range of 440–740 °C, as shown in Fig. 11. The two catalysts exhibited similar performance. At low reaction temperatures (440 °C), CH_4_ conversion was poor due to insufficient thermal energy to activate CH_4_ molecules and promote coupling reactions, resulting in a low C_2+_ yield and selectivity. Then, the catalytic performance progressively increased with temperature up to the optimum reaction temperature, which was 640 °C for the 4.6K–Co/Al_2_O_3_ catalyst and 690 °C for the 10Rb–Co/Al_2_O_3_ catalyst, after which a decline was observed. Notably, 4.6K–Co/Al_2_O_3_ consistently demonstrated higher catalytic activity than 10Rb–Co/Al_2_O_3_. At 640 °C, the optimal performance of 4.6K–Co/Al_2_O_3_ achieved a C_2+_ yield of 8.1% with 24.0% C_2+_ selectivity and 32.1% CH_4_ conversion, while the optimal performance of 10Rb–Co/Al_2_O_3_ occurred at 690 °C, resulting in a C_2+_ yield of 7.4% with 21.9% C_2+_ selectivity and 27.8% CH_4_ conversion. Above the optimum temperature, both catalysts exhibited decreased catalytic performance, likely due to the increased formation of CO and CO_2_ through the combustion of CH_4_ and C_2+_ hydrocarbons. In summary, K-doped Co/Al_2_O_3_ exhibited superior performance in C_2+_ hydrocarbon formation compared to the Rb-doped counterpart at the same molar loading.

### 3.5 Catalytic stability of the 4.6K–Co/Al_2_O_3_ catalyst for the OCM reaction

**Fig. 12:**
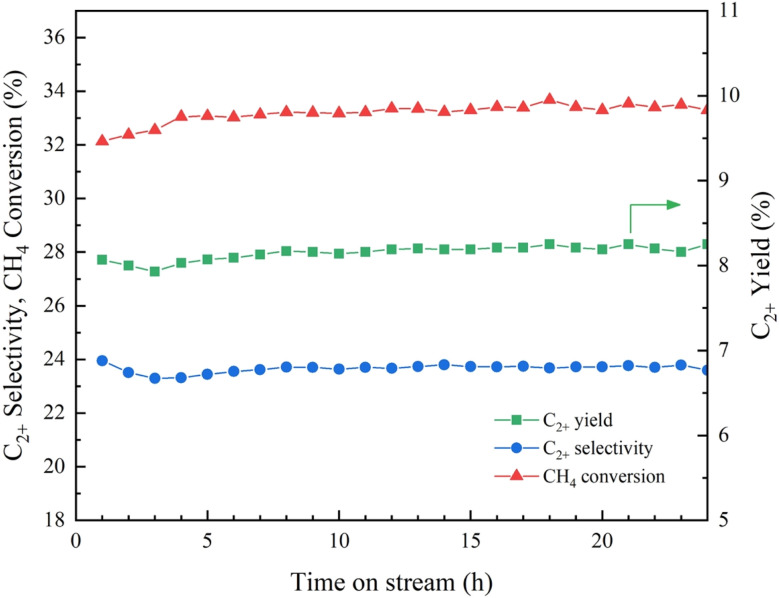
The time-on-stream performance of the 4.6K–Co/Al_2_O_3_ catalyst over 24 h. Reaction conditions: CH_4_ : O_2_ ratio = 2 : 1, catalyst weight = 40 mg, total feed flow rate = 40 mL min^−1^, reactor temperature = 640 °C.

The long-term stability of the 4.6K–Co/Al_2_O_3_ catalyst was assessed under continuous operation at 640 °C over a 24 h period, as illustrated in Fig. 12. At the beginning of the time-on-stream testing, the C_2+_ yield was 8.1%, with C_2+_ selectivity of 24.0% and a corresponding CH_4_ conversion of 32.1%. As the reaction proceeded, a gradual change in performance was observed, followed by a relatively steady. After 24 h of testing, the catalyst maintained a C_2+_ yield of 8.2%, C_2+_ selectivity of 23.6%, and CH_4_ conversion of 33.3%. According to these findings, the 4.6K–Co/Al_2_O_3_ catalyst shows excellent durability under reaction conditions, maintaining most of its initial activity over time.

The XRD analysis of the spent 4.6K–Co/Al_2_O_3_ catalyst, as shown in Fig. S2.† Peaks corresponding to Co_3_O_4_ and KNO_3_ disappeared, while new reflections attributed to the CoAl_2_O_4_ spinel phase emerged prominently. This phase transformation likely occurred due to strong interactions between Co species and the Al_2_O_3_ support under high reaction temperature conditions (640 °C) and long operation time. The analysis shown in Fig. S3† was conducted to investigate potential coke formation on the catalyst after 24 h of use. A minor signal below 100 °C is likely due to moisture evaporation. Typically, coke formation is detected by TG–DTA analysis between 200 and 600 °C.^35^ However, in this case, no such signal was observed across that range, indicating that coke did not accumulate on the catalyst surface. Although XRD patterns revealed the presence of the CoAl_2_O_4_ phase in the spent catalyst, this had minimal influence on its behavior. Overall, the TG–DTA results confirmed the absence of coke, demonstrating that the catalyst maintained excellent performance under the tested conditions.

### 3.6 *In situ* DRIFTS analysis of 4.6K–Co/Al_2_O_3_ catalyst

**Fig. 13:**
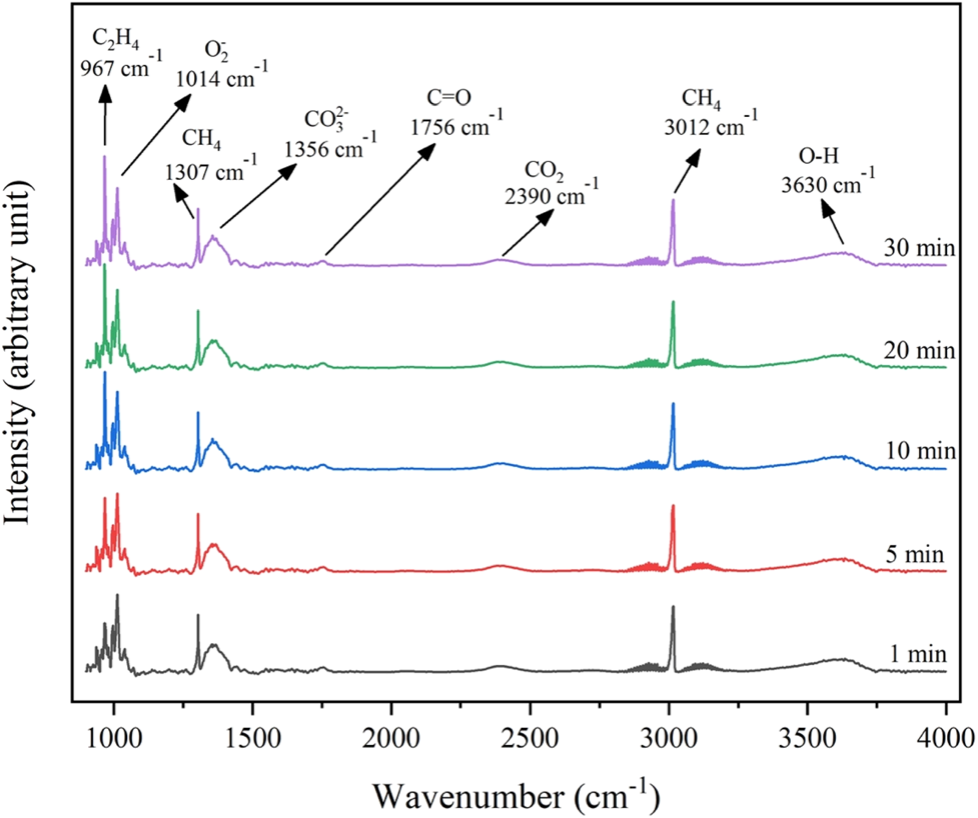
*In-situ* DRIFTS spectra of the 4.6K–Co/Al_2_O_3_ catalyst.

In the OCM reaction, the electrophilic oxygen species, including the peroxide (O_2_^2−^) and superoxide (O_2_^−^) anions, play a critical role in enhancing CH_4_ conversion and promoting C_2_ selectivity during the OCM reaction.^36^ The *in situ* DRIFTS analysis of the 4.6K–Co/Al_2_O_3_ catalyst, presented in Fig. 13, revealed a peak at 1014 cm^−1^, attributed to surface O_2_^−^ species.^30^ The peaks at 1307 and 3012 cm^−1^ correspond to the presence of CH_4_ in the gas phase, while the peak at 1356 cm^−1^ is associated with bidentate carbonate species (CO_3_^2−^).^37^ Notably, no new surface carbonate species were detected after 30 min of reaction feed exposure. This suggested that the surface O_2_^−^ species was regenerated by the O_2_ present in the reaction feed. Additionally, a peak at 967 cm^−1^ was observed, signifying the formation of C_2_H_4_ on the catalyst surface under reaction conditions.^38^ Furthermore, the catalyst had a peak at 2390 cm^−1^, characteristic of adsorbed CO_2_, and a peak at 1756 cm^−1^, attributed to C=O stretching frequency, indicating CO formation during the OCM reaction.^37^ A peak detected at 3630 cm^−1^ was assigned to the formation of O–H bonds. This suggests that a hydrogen atom from CH_4_ was chemisorbed onto reactive oxygen sites on the catalyst surface through the formation of O–H bonds.^39^ The results indicated that methane activation occurred through interaction with the active oxygen species on the 4.6K–Co/Al_2_O_3_ catalyst. This interpretation was consistent with other studies that identified oxygen as the active site for methane activation in gas–solid phase reactions.^40^

### 3.7 Proposed mechanism of the 4.6K–Co/Al_2_O_3_ catalyst for the OCM reaction

The analysis of the *in situ* DRIFTS results in Fig. 13, combined with insights from other studies on catalysts used in the OCM reaction, provided essential information for understanding the catalytic mechanism of the 4.6K–Co/Al_2_O_3_ catalyst. Initially, molecular O_2_ dissociated on the catalyst surface, producing O_2_^−^ species, which appeared at 1014 cm^−1^ in Fig. 13. It is also noted that the O_2_^−^ band's constant intensity indicated that the consumption and regeneration of O_2_^−^ proceed at a sufficiently rapid rate to achieve equilibrium at the reaction temperature.^36^ Then, the O_2_^−^ species extracted hydrogen from CH_4_, forming methyl radicals (˙CH_3_) and surface hydroxyl groups (—OH),^39^ which was confirmed by the *in situ* DRIFTS peak at 3630 cm^−1^. The methyl radicals combined in the gas phase to form C_2_H_6_, which can subsequently produce C_2_H_4_, *via* dehydrogenation processes.^41^ The synthesis of C_2_H_4_ on the catalyst surface was indicated by the peak at 967 cm^−1^, with the concentration increasing with reaction time. The adsorbed –OH species may desorb from the surface as either ˙OH or ˙H radicals, which can further react to form H_2_O. In addition, uncoupled radicals and hydrocarbons may undergo additional oxidation, forming CO and CO_2_,^42^ which appeared at 1756 and 2390 cm^−1^, respectively.

However, a comparison between the mechanistic pathway proposed in this study and that of previously reported hybrid systems—specifically, the dual-layer catalyst comprising 5Ni/Al_2_O_3_ as the first layer and 4.6K–Co/Al_2_O_3_ as the second—reveals a fundamental distinction: in the earlier system, the reaction initiates as the reactant gases (CH_4_ and O_2_) pass through the first layer of the hybrid catalyst system, a portion of CH_4_ is transformed into CO, CO_2_, and H_2_*via* the partial oxidation of methane (POM) reaction. These products, particularly CO and H_2_, serve as intermediate species for the subsequent Fischer–Tropsch synthesis occurring over the second catalyst layer. In the Fischer–Tropsch mechanism, which follows a chain-growth polymerization model, syngas components (CO and H_2_) undergo surface dissociation into C, O, and H atoms during the initiation phase. A surface-bound C atom subsequently reacts with H atoms to generate CH_2_ monomers, which then polymerize through successive coupling steps, ultimately leading to the formation of longer-chain hydrocarbons.

The 4.6K–Co/Al_2_O_3_ catalyst has several key components, each contributing to the catalytic process. The active components of this catalyst include K, Co, and Al_2_O_3_. The K component serves as a promoter, enhancing the number of basic sites on the catalyst that are essential for forming C_2+_ hydrocarbons.^43^ Cobalt oxides, particularly Co_3_O_4_ with a spinel structure, are highly efficient at methane adsorption. Additionally, cobalt-based catalysts have major activity and selectivity in producing long-chain hydrocarbons.^44^ Primarily, Al_2_O_3_ serves as a support material for the catalyst, owing to its advantageous properties, including a high surface area and well-distributed pore sizes, which facilitate superior metal dispersion and enhance catalyst stability.^45^ In the current study, these factors likely contributed to the catalyst's high activity and exceptional performance at relatively low temperatures during the OCM reaction.

### 3.8 Comparative performance of 4.6K–Co/Al_2_O_3_ with other catalysts

**Fig. 14:**
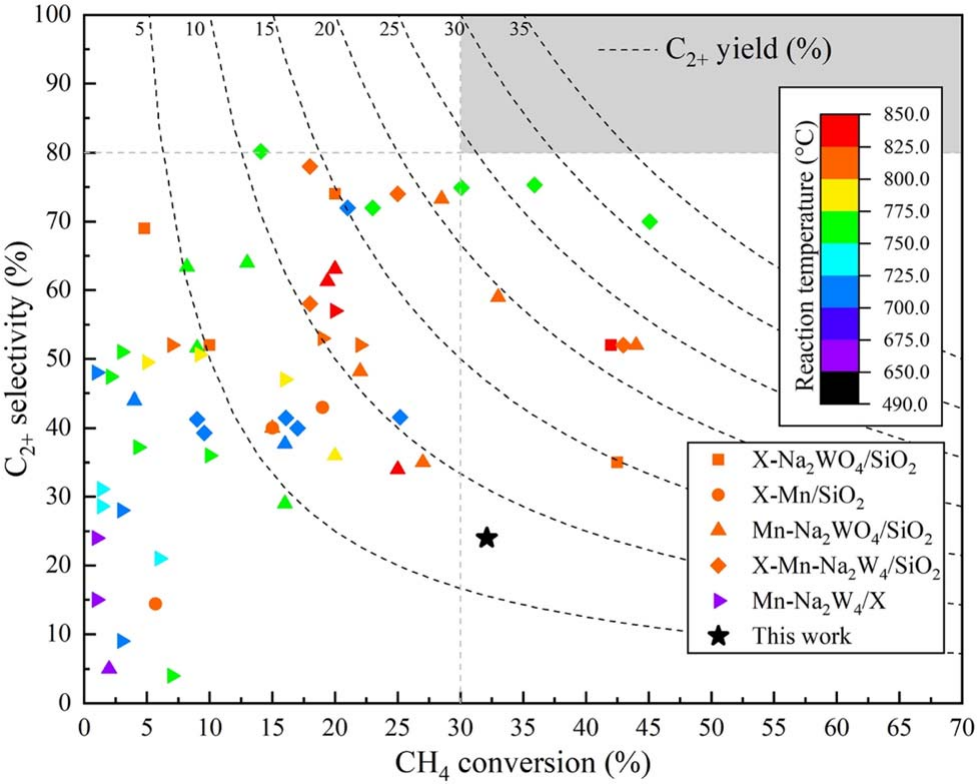
Comparison of the catalyst developed in this study with other catalysts previously reported for the OCM reaction.

Several catalysts investigated previously—particularly those comprising Na_2_WO_4_ in combination with Mn—have been recognized for their superior reactivity in the OCM process. To evaluate the performance of the optimized K–Co/Al_2_O_3_ catalyst developed in this study, a comparative analysis was conducted against selected representatives from this category, as illustrated in Fig. 14 and detailed in Table S3.† Reported performance for Na_2_WO_4_–Mn catalysts varies widely, with C_2+_ yields ranging from 0.2% to 31.6%, C_2+_ selectivities between 4.0% and 80.2%, and CH_4_ conversion 1.0% to 45.4% at reaction temperatures of 650–850 °C. However, achieving both high conversion and high selectivity concurrently remains challenging. For commercial viability, a benchmark of at least 30% CH_4_ conversion and 80% C_2+_ selectivities is typically required (as highlighted by the gray zone in Fig. 14). While a few catalysts exceed one of these thresholds, simultaneous attainment is rarely observed, indicating limitations in current materials. Notably, the K–Co/Al_2_O_3_ catalyst presented in this work attained 32.1% CH_4_ conversion with 24.0% selectivity and 8.1%C_2+_ yield products at 640 °C, which lower than the operational temperatures of many high-performing systems. These findings underscore the importance of developing next-generation catalysts that can deliver high selectivity (>80%) while maintaining efficient CH_4_ conversion (>30%), which is a critical direction for future innovation in OCM catalysis.

## 4. Conclusion

This study demonstrates the considerable influence of alkali metal promotion on the catalytic performance of Co/Al_2_O_3_ for the oxidative coupling of methane (OCM), a process crucial for sustainable methane utilization. Among the investigated alkali metal promoters (Li, Na, K, and Rb), K-promoted catalysts produced the most pronounced enhancement, with the optimized 4.6K–Co/Al_2_O_3_ catalyst achieving 8.1% C_2+_ yield, 24.0% C_2+_ selectivity, and 32.1% CH_4_ conversion at 640 °C. Characterization revealed that K increased the surface basicity and modified the electronic environment of active sites, facilitating selective methane activation and suppressing complete oxidation to CO and CO_2_. The mechanistic investigations, supported by *in situ* DRIFTS analysis, demonstrated that molecular O_2_ dissociated on the catalyst, generating O_2_^−^ species that extracted hydrogen from CH_4_ to generate surface –OH groups and ˙CH_3_, which subsequently recombined to produce C_2_H_6_ and dehydrogenated into C_2_H_4_. While Rb demonstrated potential, Li and Na had comparatively lower efficacy, emphasizing the importance of promoter selection in optimizing catalytic performance. This research established that alkali metal-promoted Co/Al_2_O_3_, particularly the K-promoted variant, was a promising candidate for low-temperature OCM applications. These findings should provide valuable insights into the design of efficient, selective, and stable catalysts for the valorization of methane. Future research should focus on improving the properties of the catalyst and enhancing its performance to realize the full industrial potential of these systems.

## Author contributions

Sarannuch Sringam: Writing – original draft, writing – review & editing, methodology, investigation, validation, formal analysis, data curation. Punyanut Thansiriphat: Methodology, investigation. Thongthai Witoon: Methodology, investigation conceptualization. Waleeporn Donphai: Methodology, investigation. Metta Chareonpanich: Resources, conceptualization. Chularat Wattanakit: Resources, methodology. Hiesang Sohn: Resources, review & editing. Nevzat Yigit: Resources, methodology. Günther Rupprechter: Resources, conceptualization. Anusorn Seubsai: Writing – original draft, writing – review & editing, methodology, investigation, validation, formal analysis, data curation, supervision, project administration.

## Conflicts of interest

There are no conflicts to declare.

## Supplementary Material

RA-015-D5RA02408K-s001

## Data Availability

The datasets used and/or analyzed during the current study are available from the corresponding author upon reasonable request.
