# Supplementary material for: Direct conversion of methane to value-added hydrocarbons using alkali metal-promoted cobalt catalysts
Source: RSC Adv. 2025 Jul 7;15(28):23103–14. doi: 10.1039/d5ra02408k (PMC12230545; doi:10.1039/d5ra02408k)
Supplement: RA-015-D5RA02408K-s001 [file RA-015-D5RA02408K-s001.pdf]

Supplementary Information (SI) for RSC Advances.
This journal is © The Royal Society of Chemistry 2025

Supplementary Information

Direct conversion of methane to value-added hydrocarbons using alkali metal-promoted cobalt catalysts

Sarannuch Sringam[1,2], Punyanut Thansiriphat, Thongthai Witoon[1,2], Waleeporn Donphai[1], Metta Chareonpanich[1,2], Chularat Wattanakit[3], Hiesang Sohn[4], Nevzat Yigit[5], Günther Rupprechter[5], Anusorn Seubsai[1,2]*

[1] *Department of Chemical Engineering, Faculty of Engineering, Kasetsart University, Bangkok 10900, Thailand*

[2] *Center of Excellence on Petrochemical and Materials Technology, Kasetsart University, Bangkok 10900, Thailand*

[3] *Department of Chemical and Biomolecular Engineering, School of Energy Science and Engineering, Vidyasirimedhi Institute of Science and Technology, Rayong 21210, Thailand*

[4] *Department of Chemical Engineering, Kwangwoon University, Seoul, 01897, South Korea*

[5] *Institute of Materials Chemistry, Technische Universität Wien, Vienna 1060, Austria*

***Corresponding author**: fengasn@ku.ac.th

Table S1

The crystalline structure of catalysts

Catalyst	Crystalline phase	2θ (degree)	ICDD No.
Co/Al_2O_3	Co_3O_4	18.99, 31.26, 36.84, 38.54, 44.80, 55.64, 59.34, 65.22, 78.38	01-076-1802
	γ-Al_2O_3	39.48, 45.91, 66.95	01-075-0921
4.6K-Co/Al_2O_3	Co_3O_4	19.10, 31.39, 36.73, 38.73, 44.93, 55.82, 59.52, 65.40, 78.62	01-076-1802
	KNO_3	27.40, 32.86, 39.57, 44.93, 67.11	01-078-7937
	γ-Al_2O_3	39.57, 46.44, 67.11	01-075-0921
4.6Rb-Co/Al_2O_3	Co_3O_4	19.03, 31.32, 36.90, 38.61, 44.88, 55.75, 59.45, 65.34, 78.54	01-076-1802
	γ-Al_2O_3	39.48, 45.95, 66.95	01-075-0921

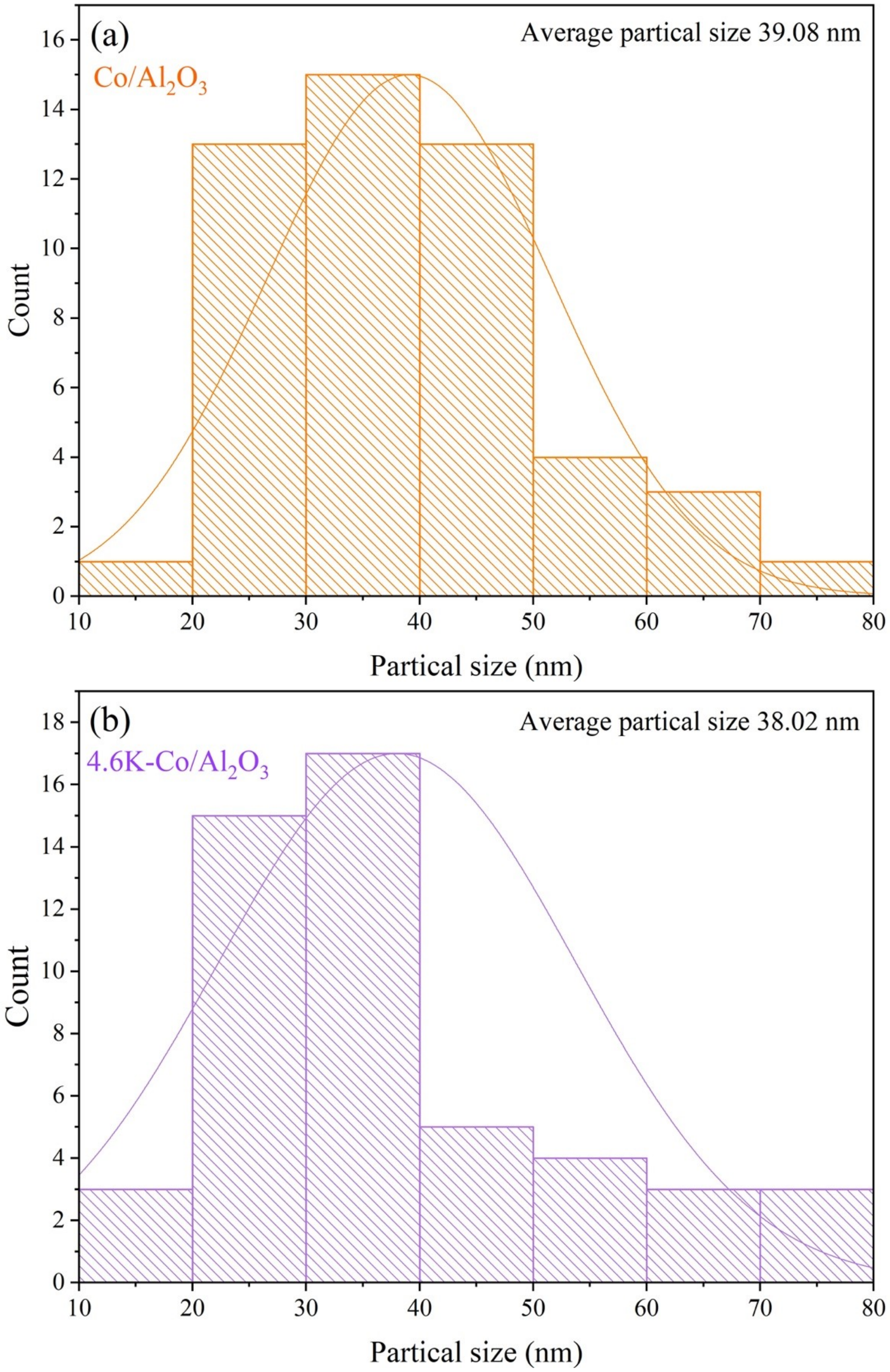
(a)
Co/Al2O3
Average partical size 39.08 nm
Count
Partical size (nm)
(b)
4.6K-Co/Al2O3
Average partical size 38.02 nm
Count
Partical size (nm)

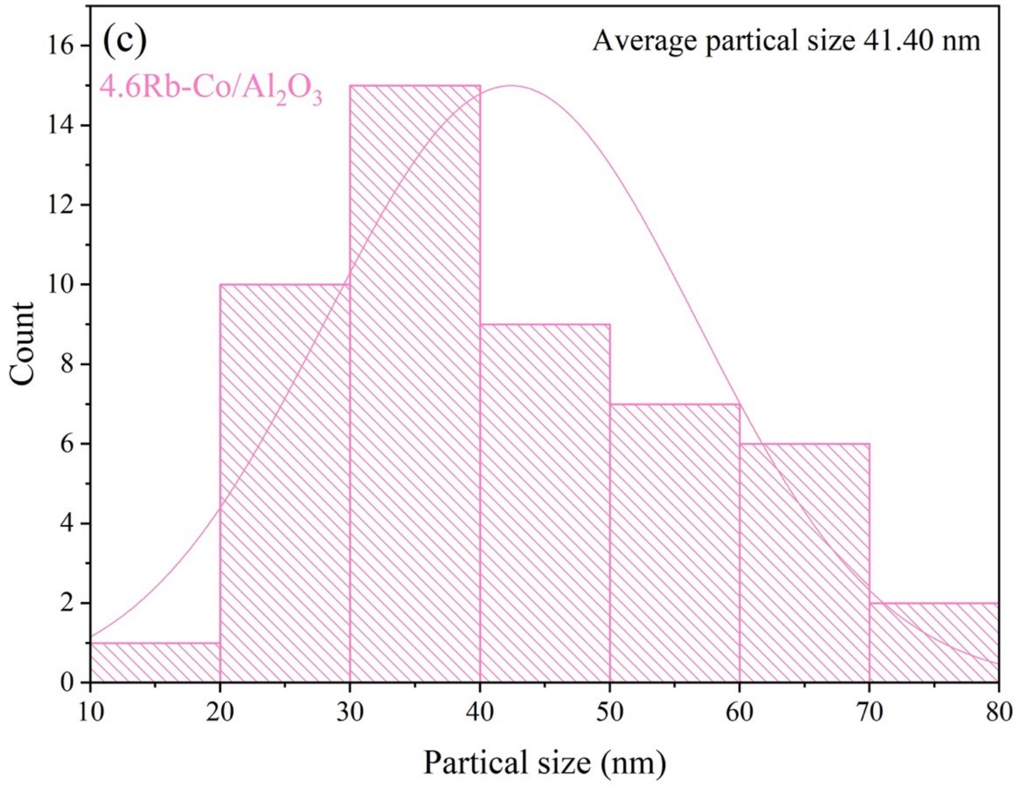

Fig. S1. The particle size distribution of (a) Co/Al_2O_3, (b) 4.6K-Co/Al_2O_3, and (c) 4.6Rb-Co/Al_2O_3 catalysts.

Table S2

Summary XPS peaks of Co 2p of the catalysts

Catalyst	Binding Energy (eV)			
	Co $2p^{3/2}$	1st satellite	Co $2p^{1/2}$	2nd satellite
Co/Al_2O_3	781.1	784.1	796.1	798.9
$4.6K\text{-}Co/Al_2O_3$	780.5	783.0	795.2	796.6
$4.6Rb\text{-}Co/Al_2O_3$	780.8	783.1	795.7	797.4

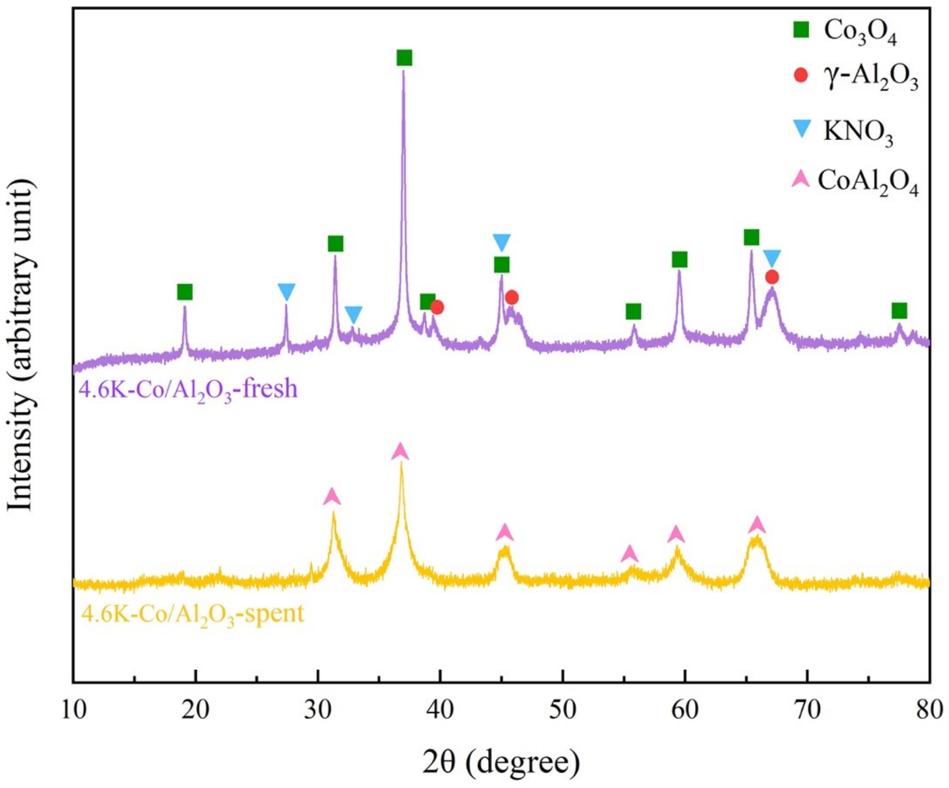

Fig. S2. XRD patterns of fresh and spent 4.6K-Co/Al_2O_3 catalyst.

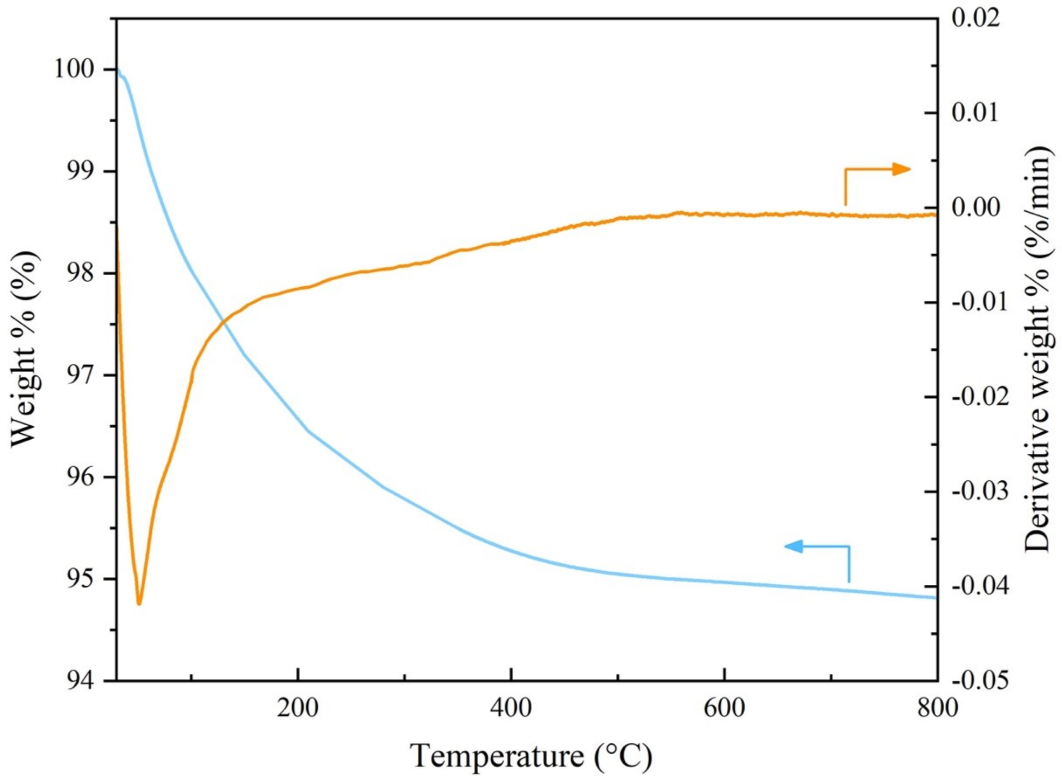

Fig. S3. Thermogravimetric analysis profile of weight loss and derivative of weight loss in N_2 atmosphere of spent 4.6K-Co/Al_2O_3 catalyst.

Table S3

A survey of catalysts reported in literature.

Catalyst	C_{2+} yield (%)	C_{2+} selectivity (%)	CH_4 conversion (%)	Reaction temperature (°C)	Ref.
Na_2WO_4-TiO_2-MnO/SiO_2	6.7	41.5	16.1	700	[1]
Li-Na_2WO_4-TiO_2-MnO/SiO_2	3.8	39.3	9.6	700	[1]
K-Na_2WO_4-TiO_2-MnO/SiO_2	6.8	40.0	17.02	700	[1]
Rb-Na_2WO_4-TiO_2-MnO/SiO_2	10.5	41.6	25.2	700	[1]
Cs-Na_2WO_4-TiO_2-MnO/SiO_2	3.7	41.3	9.0	700	[1]
Li-W/SiO_2	6.0	52.0	10.0	800	[2]
Li-Mn/SiO_2	6.0	40.0	15.0	800	[2]
Mn-W/SiO_2	6.0	40.0	15.0	800	[2]
Mn-Li-W/SiO_2	11.0	58.0	18.0	800	[2]
Mn-Li-W/SiO_2	15.0	78.0	18.0	800	[2]
Na-W-Mn/SiO_2	24.0	52.0	44.0	800	[3]
Li-Na-W-Mn/SiO_2	22.0	52.0	43.0	800	[3]

Catalyst	C_{2+} yield (%)	C_{2+} selectivity (%)	CH_4 conversion (%)	Reaction temperature (°C)	Ref.
Na_2WO_4/SiO_2	19.0	52	44	850	[4]
K_2WO_4/SiO_2	16.0	67	30	850	[4]
Rb_2WO_4/SiO_2	19	78	32	850	[4]
Cs_2WO_4/SiO_2	13	55	33	850	[4]
Li_2WO_4/SiO_2	9.5	27	36	850	[4]
Mn/SiO_2	0.6	14.4	5.7	800	[5]
Na_2WO_4/SiO_2	2.9	69.0	4.8	800	[5]
$Mn-Na_2WO_4/SiO_2$	18.5	73.3	28.5	800	[5]
Na_2WO_4/SiO_2	15	74	20	800	[6]
Mn/SiO_2	9	43	19	800	[6]
$Mn-Na_2WO_4/SiO_2$	6.0	37.7	16.0	700	[7]
$Mn-Na_2WO_4/SiO_2$	4.6	51.6	9.0	750	[7]
$Mn-Na_2WO_4/SiO_2$	10.6	48.2	22.0	800	[7]
$TiO_2-Mn-Na_2WO_4/SiO_2$	15.1	72	21	720	[8]
$TiO_2-Mn-Na_2WO_4/SiO_2$	16.6	72	23	760	[8]
$TiO_2-Mn-Na_2WO_4/SiO_2$	18.5	74	25	800	[8]
$Mn-Na_2WO_4/SiO_2$	10	29	16	770	[9]
$Mn-Na_2WO_4/SiO_2$	14	36	20	790	[9]

Catalyst	C_{2+} yield (%)	C_{2+} selectivity (%)	CH_4 conversion (%)	Reaction temperature (°C)	Ref.
$Mn\text{-}Na_2WO_4/SiO_2$	16	35	27	810	[9]
$Mn\text{-}Na_2WO_4/SiO_2$	15	34	25	830	[9]
$Mn\text{-}Na_2WO_4/SiO_2$	12.6	63.1	20.0	840	[9]
$Mn\text{-}Na_2WO_4/SiO_2$	1.9	61.3	19.4	840	[9]
$Mn–Na_2WO_4/SiO_2$	5.2	63.4	8.2	750	[10]
(NaCl+KCl)–Mn Na_2WO_4/SiO_2	31.6	70.0	45.1	750	[10]
$KCl–Mn–Na_2WO_4/SiO_2$	27.0	75.3	35.9	750	[10]
$CsCl\text{-}Mn\text{-}Na_2WO_{44}/SiO_2$	22.6	74.9	30.1	750	[10]
$LiCl\text{-}Mn\text{-}Na_2WO_4/SiO_2$	11.3	80.2	14.1	750	[10]
$Mn\text{-}Na_2WO_4/SiO_2$	1.0	5.0	2.0	650	[11]
$Mn\text{-}Na_2WO_4/SiO_2$	1.8	44.0	4.0	700	[11]
$Mn\text{-}Na_2WO_4/SiO_2$	8.3	64.0	13.0	750	[11]
$Mn\text{-}Na_2WO_4/SiO_2$	19.5	59.0	33.0	800	[11]
$Mn\text{-}Na_2WO_4/SiC$	0.2	15.0	1.0	650	[11]
$Mn\text{-}Na_2WO_4/SiC$	0.8	28.0	3.0	700	[11]
$Mn\text{-}Na_2WO_4/SiC$	0.2	4.0	7.0	750	[11]
$Mn\text{-}Na_2WO_4/SiC$	11.4	52.0	22.0	800	[11]

Catalyst	C_{2+} yield (%)	C_{2+} selectivity (%)	CH_4 conversion (%)	Reaction temperature (°C)	Ref.
Mn-Na_2WO_4/SiO_2-SiC	0.2	24.0	1.0	650	[11]
Mn-Na_2WO_4/SiO_2-SiC	0.5	48.0	1.0	700	[11]
Mn-Na_2WO_4/SiO_2-SiC	1.5	51.0	3.0	750	[11]
Mn-Na_2WO_4/SiO_2-SiC	3.6	52.0	7.0	800	[11]
Mn-Na_2WO_4/Al_2O_3	0.27	9.0	3.0	700	[12]
Mn-Na_2WO_4/Al_2O_3	1.26	21.0	6.0	725	[12]
Mn-Na_2WO_4/Al_2O_3	3.6	36.0	10.0	750	[12]
Mn-Na_2WO_4/Al_2O_3	7.52	47.0	16.0	775	[12]
Mn-Na_2WO_4/Al_2O_3	10.07	53.0	19.0	800	[12]
Mn-Na_2WO_4/Al_2O_3	11.4	57.0	20.0	825	[12]
Mn-Na_2WO_4/Al_2O_3	11.4	57.0	20.0	850	[12]
Mn-Na_2WO_4/Al_2O_3	12.3	19.5	63.0	800	[13]
Mn-Na_2WO_4/Al_2O_3	18.5	29.0	63.8	825	[13]
Mn-Na_2WO_4/Al_2O_3	19.4	30.0	64.5	850	[13]
Mn-Na_2WO_4/Al_2O_3-TiO_2	16.0	25.0	63.8	800	[13]
Mn-Na_2WO_4/Al_2O_3-TiO_2	15.4	24.5	63.0	825	[13]
Mn-Na_2WO_4/Al_2O_3-TiO_2	11.4	19.0	60.2	850	[13]
Mn-Na_2WO_4/Al_2O_3-SiO_2	14.6	23.0	63.4	800	[13]

Catalyst	C_{2+} yield (%)	C_{2+} selectivity (%)	CH_4 conversion (%)	Reaction temperature (°C)	Ref.
Mn-Na_2WO_4/Al_2O_3-SiO_2	13.9	22.0	63.0	825	[13]
Mn-Na_2WO_4/Al_2O_3-SiO_2	9.0	14.5	61.9	850	[13]
Na/W/Mn/MgO	0.4	31.1	1.4	725	[14]
Na/W/Mn/MgO	1.6	37.2	4.3	750	[14]
Na/W/Mn/MgO	4.6	50.6	9.2	775	[14]
Na/W/Mn/TiO_2	0.4	28.6	1.4	725	[14]
Na/W/Mn/TiO_2	1.0	47.4	2.1	750	[14]
Na/W/Mn/TiO_2	2.4	49.5	5.0	775	[14]

Reference

1. Kidamorn, P., et al., *Synthesis of Value-Added Chemicals via Oxidative Coupling of Methanes over Na_2WO_4–TiO_2–MnO_x/SiO_2 Catalysts with Alkali or Alkali Earth Oxide Additives.* ACS Omega, 2020. **5**(23): p. 13612-13620.
2. Malekzadeh, A., et al., *Oxidative Coupling of Methane over Lithium Doped (Mn+W)/SiO_2 Catalysts.* Journal of Natural Gas Chemistry, 2007. **16**(2): p. 121-129.
3. Nipan, G.D., et al., *Isovalent Substitutions in Composite Catalysts Na/W/Mn/SiO_2.* Russian Journal of Inorganic Chemistry, 2019. **64**(9): p. 1115-1119.
4. Palermo, A., J.P. Holgado Vazquez, and R.M. Lambert, *New efficient catalysts for the oxidative coupling of methane.* Catalysis Letters, 2000. **68**(3): p. 191-196.
5. Elkins, T.W. and H.E. Hagelin-Weaver, *Characterization of Mn–Na_2WO_4/SiO_2 and Mn–Na_2WO_4/MgO catalysts for the oxidative coupling of methane.* Applied Catalysis A: General, 2015. **497**: p. 96-106.
6. Gholipour, Z., et al., *Oxidative coupling of methane over (Na_2WO_4+Mn or Ce)/SiO_2 catalysts: In situ measurement of electrical conductivity.* Journal of Natural Gas Chemistry, 2010. **19**(1): p. 35-42.
7. Ortiz-Bravo, C.A., et al., *Elucidating the structure of the W and Mn sites on the Mn-Na_2WO_4/SiO_2 catalyst for the oxidative coupling of methane (OCM) at real reaction temperatures.* Journal of Catalysis, 2022. **408**: p. 423-435.
8. Wang, P., et al., *TiO_2-doped Mn_2O_3-Na_2WO_4/SiO_2 catalyst for oxidative coupling of methane: Solution combustion synthesis and $MnTiO_3$-*

dependent low-temperature activity improvement. Applied Catalysis A: General, 2017. **544**.

9. Koirala, R., et al., *Oxidative coupling of methane on flame-made Mn-Na_2WO_4/SiO_2: Influence of catalyst composition and reaction conditions.* Applied Catalysis A: General, 2014. **484**: p. 97-107.
10. Hiyoshi, N. and T. Ikeda, *Oxidative coupling of methane over alkali chloride–Mn–Na_2WO_4/SiO_2 catalysts: Promoting effect of molten alkali chloride.* Fuel Processing Technology, 2015. **133**: p. 29-34.
11. Lezcano, G., et al., *Effect of the particle blending-shaping method and silicon carbide crystal phase for Mn-Na-W/SiO_2-SiC catalyst in oxidative coupling of methane.* Molecular Catalysis, 2022. **527**: p. 112399.
12. L'hospital, V., et al., *Oxidative coupling of biogas to ethylene over a trilobe-shaped Mn-Na_2WO_4/α-Al_2O_3 catalyst in a single-pellet reactor.* Applied Catalysis A: General, 2023. **666**: p. 119402.
13. Alahmadi, F., et al., *Oxidative coupling of methane over strontium-doped neodymium oxide: Parametric evaluations.* AIChE Journal, 2023. **69**(4): p. e17959.
14. Jeon, W., et al., *Oxidative coupling of methane to C_2 hydrocarbons on the Mg–Ti mixed oxide-supported catalysts at the lower reaction temperature: Role of surface oxygen atoms.* Applied Catalysis A: General, 2013. **464-465**: p. 68-77.
